# TTK Inhibition Alleviates Postinjury Neointimal Formation and Atherosclerosis

**DOI:** 10.1002/advs.202409250

**Published:** 2024-12-24

**Authors:** Jie‐Hong Wu, Yu‐Xiao Liu, Jia‐Bin Zong, Min Qiu, Yi‐Fan Zhou, Ya‐Nan Li, Tuersun Aili, Xin‐Ran Zhao, Bo Hu

**Affiliations:** ^1^ Department of Neurology Union Hospital Tongji Medical College Huazhong University of Science and Technology Wuhan 430022 China

**Keywords:** atherosclerosis, p120‐catenin, restenosis, TTK, vascular smooth muscle cells

## Abstract

Atherosclerosis and its associated cardio‐cerebrovascular complications remain the leading causes of mortality worldwide. Current lipid‐lowering therapies reduce only approximately one‐third of the cardiovascular risk. Furthermore, vascular restenosis and thrombotic events following surgical interventions for severe vascular stenosis significantly contribute to treatment failure. This highlights the urgent need for novel therapeutic targets to manage atherosclerosis and prevent restenosis and thrombosis after vascular injury. This study identifies TTK protein kinase (TTK) as a key regulator of vascular smooth muscle cell (VSMC) phenotypic switching in the context of postinjury neointimal formation and atherosclerosis. Mechanistically, TTK upregulation in VSMCs phosphorylates p120‐catenin, leading to β‐catenin nuclear accumulation and dissociation of the myocardin (MYOCD)/serum response factor (SRF) complex. Deletion of TTK specifically in VSMCs reduces postinjury neointimal formation in vascular injury models and attenuates atherosclerotic lesions in *ApoE^−/−^* mice. Notably, oral administration of the TTK inhibitor CFI‐402257 mitigated neointimal formation without impairing reendothelialization and reduced atherosclerotic lesions in *ApoE^−/−^* mice without altering lipid levels. These findings suggest that targeting TTK, through inhibitors or alternative strategies, represents a promising approach to simultaneously prevent postinjury restenosis and treat atherosclerosis.

## Graphical abstract

This study identifies TTK as a key inducer of vascular smooth muscle cell (VSMC) phenotypic switching in the context of postinjury neointimal formation and atherosclerosis. Mechanistically, TTK upregulation in VSMCs phosphorylates p120‐catenin, leading to β‐catenin nuclear accumulation and dissociation of the myocardin/serum response factor complex. Targeting TTK represents a promising approach to simultaneously prevent postinjury restenosis and treat atherosclerosis.

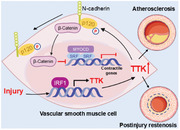

## 1 Introduction

Atherosclerosis and its associated cardio‐cerebrovascular complications, such as myocardial infarction and stroke, are the leading causes of mortality worldwide.^[^ ^1^ ^]^ The development of atherosclerotic plaques disrupts blood flow to the heart and brain, leading to severe clinical symptoms. Current therapeutic strategies primarily rely on lipid‐lowering agents, including statins, ezetimibe, and PCSK9 inhibitors.^[^ ^2^ ^]^ Despite their efficacy, these therapies reduce only approximately one‐third of the cardio‐cerebrovascular risk, leaving many patients vulnerable to atherosclerotic plaque progression. Additionally, for patients with severe vascular stenosis, surgical interventions such as balloon angioplasty, stent implantation, bypass grafting or endarterectomy are critical complementary treatments. However, restenosis and lumen thrombotic events following these procedures remain significant challenges, often resulting in treatment failure.^[^ ^3^, ^4^, ^5^ ^]^ Thus, there is an urgent need for innovative approaches to treat atherosclerosis and mitigate postinjury restenosis and thrombotic events.

Vascular smooth muscle cells (VSMCs), the predominant components of the arterial wall, typically exhibit a contractile phenotype that maintains vascular homeostasis. Previous studies report that VSMCs undergo pathological phenotypic switching, transitioning from a contractile to a synthetic phenotype characterized by increased proliferation and migration, which contributes to neointima formation during atherosclerosis and postinjury restenosis.^[^ ^6^, ^7^, ^8^ ^]^ Despite advancement in understanding VSMC biology, the molecular drivers of this pathological phenotypic switching remain incompletely elucidated. Additionally, surgical–induced vascular injury requires an efficient reendothelialization to prevent thrombotic events,^[^ ^9^, ^10^, ^11^ ^]^ but current drug‐eluting stents often inhibit both VSMC phenotypic switching and reendothelialization, increasing the risk of lumen thrombotic events. This highlights the need for new targets that can selectively inhibit VSMC phenotypic switching without impairing reendothelialization, offering a dual benefit in treating atherosclerosis and preventing restenosis.

TTK (TTK Protein Kinase), a dual‐specific protein kinase, can phosphorylate tyrosine, serine, and threonine.^[^ ^12^ ^]^ Previous studies have indicated that TTK is typically undetectable in normal cells but is significantly upregulated in various cancers.^[^ ^13^, ^14^, ^15^, ^16^, ^17^ ^]^ Additionally, TTK promotes the metastatic potential of cancer cells, contributing to tumor progression and poor prognosis in several cancers.^[^ ^18^, ^19^ ^]^ However, its role in atherosclerosis and postinjury restenosis has not been previously investigated.

By re‐analyzing transcriptomic datasets from the Gene Expression Omnibus (GEO), this study identified TTK as a novel regulator of VSMC phenotypic switching in the contexts of postinjury neointimal formation and atherosclerosis. Mechanistically, TTK upregulation in VSMCs phosphorylated p120‐catenin, triggering β‐catenin nuclear accumulation and myocardin (MYOCD)/serum response factor (SRF) complex dissociation, thereby promoting VSMC phenotypic switching. Smooth muscle cell‐specific knockout of TTK alleviated both postinjury neointima formation in vascular injury models and reduced atherosclerotic lesions in *ApoE^−/−^* mice without altering lipid profiles. Moreover, oral administration of a TTK inhibitor simultaneously exerted inhibitory effects on postinjury neointimal formation and atherosclerosis while preserving reendothelialization. Thus, targeting TTK using inhibitors or alternative strategies could be effective to simultaneously prevent postinjury restenosis and treat atherosclerosis.

## 2 Results

### 2.1 TTK is Upregulated During Neointima Formation in Vascular Injury and Atherosclerosis

**Figure 1:**
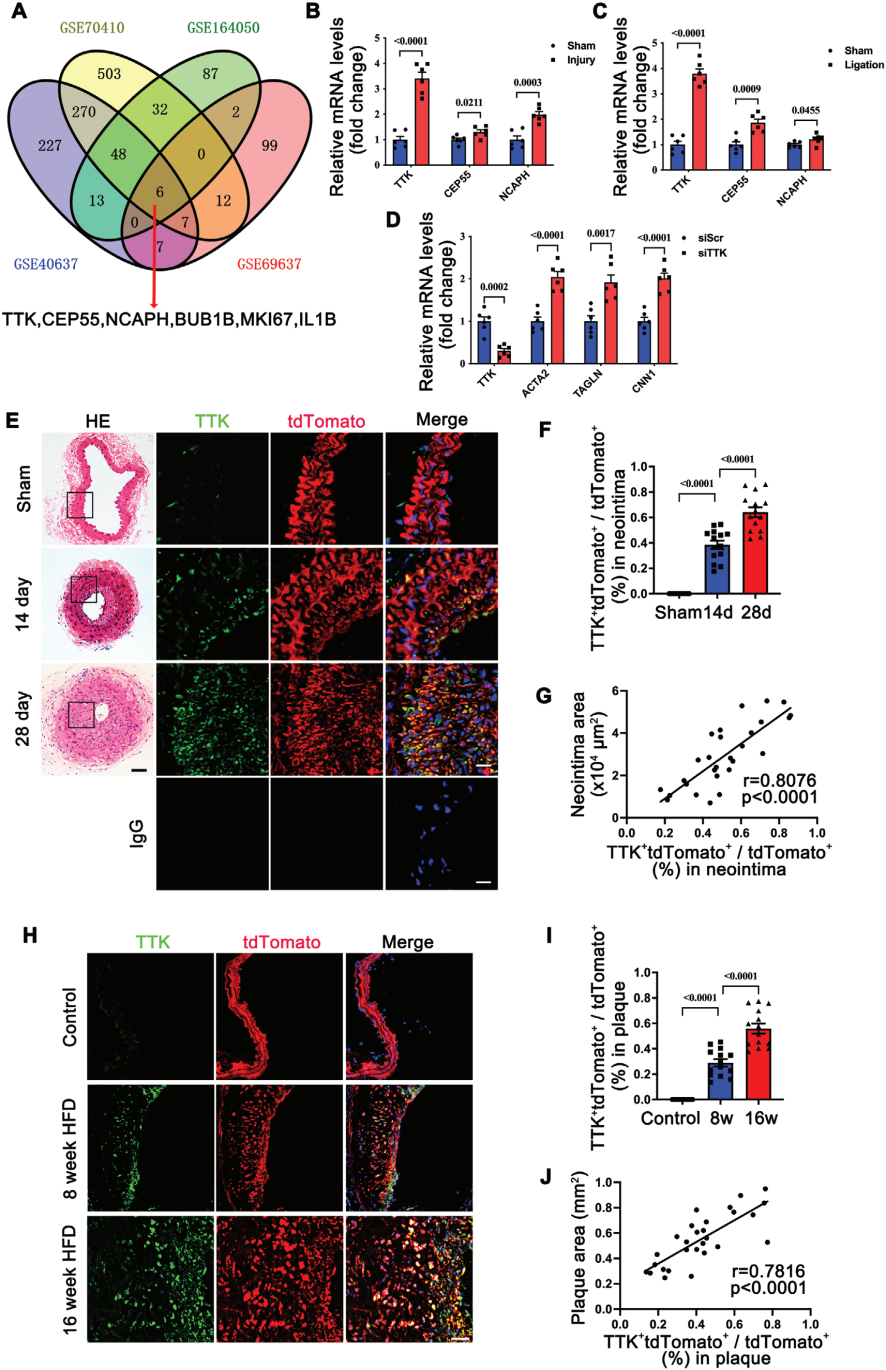
TTK is upregulated during neointima formation in vascular injury and atherosclerosis. A) Venn diagram of the differentially expressed genes (DEGs) in four Gene Expression Omnibus (GEO) datasets was used to identify novel molecules involved in the phenotypic switching of VSMCs under different pathological conditions. B) Quantitative real‐time polymerase chain reaction (qRT‐PCR) validation of the expression of novel VSMC phenotype‐related genes in sham‐operated or wire‐injured carotid arteries of C57 mice on day 28 post‐surgery (*n* = 6). C) qRT‐PCR validation of the expression of novel VSMC phenotype‐related genes in sham‐operated or ligated carotid arteries of C57 mice on day 28 post‐surgery (*n* = 6). D) qRT‐PCR analysis of the relative mRNA level of TTK, ACTA2, TAGLN, and CNN1 in VSMCs transfected with scrambled siRNA or TTK‐specific siRNA (*n* = 6). E) Representative hematoxylin and eosin (HE) staining (left) and immunofluorescence (right) staining of TTK (green) and tdTomato (red) in the sham‐operated or wire‐injured carotid sections of *Myh11‐CreER^T2^*/*Rosa26^tdTomato^* mice on days 14 and 28 post‐surgery. IgG was used as a negative control. Nuclei were stained with 4′,6‐diamidino‐2‐phenylindole (DAPI) (blue). Scale bar = 50 µm (left) or 10 µm (right). F) The percentage of TTK‐positive, tdTomato‐positive VSMCs in the neointima (*n* = 14). G) Analysis of the correlation between the neointima area and the percentage of TTK‐positive, tdTomato‐positive VSMCs in neointima (*n* = 28). H) Representative immunofluorescence staining of TTK (green) and tdTomato (red) in the aortic root sections of *Myh11‐CreER^T2^*/*Rosa26^tdTomato^*/*ApoE^−/−^* mice fed on a high‐fat diet (HFD) for 0, 8, and 16 weeks. Nuclei were stained with DAPI (blue). Scale bar = 50 µm. I) The percentage of TTK‐positive, tdTomato‐positive VSMCs in atherosclerotic plaques (*n* = 14). J) Analysis of the correlation between plaque area and the percentage of TTK‐positive, tdTomato‐positive VSMCs in atherosclerotic plaques (*n* = 28). Data are presented as the mean ± SEM; unpaired *t*‐test, one‐way ANOVA.

To identify novel molecules involved in VSMC phenotypic switching during neointima formation, we reanalyzed differentially expressed genes (DEGs) from four publicly available transcriptomic datasets (Data S1, Supporting Information). These datasets included models of wire injury, carotid ligation, balloon injury, and in vitro PDGF‐BB‐stimulated VSMCs. Overlapping DEGs revealed six genes (TTK, CEP55, NCAPH, BUB1B, MKI67, and IL1B) consistently upregulated across all pathological conditions (**Figure** **1**A). Of these, TTK, CEP55, and NCAPH had not previously been associated with VSMC phenotypic switching. To validate these findings, we assessed the mRNA levels of TTK, CEP55, and NCAPH in mouse models of wire‐injured and ligated carotid arteries. TTK showed the most pronounced upregulation in both conditions (Figure 1B,C). Further, silencing TTK, CEP55, and NCAPH using specific siRNAs in mouse VSMCs demonstrated that TTK knockdown significantly increased the expression of VSMC contractile markers, including α‐SMA, SM22α, and Calponin1 (encoded by ACTA2, TAGLN, and CNN1) (Figures 1D and S1A,B, Supporting Information). These findings identified TTK as a key focus for further exploration in VSMC phenotypic switching during neointima formation.

Given the previously uncharacterized role of TTK in the vascular system, we next examined its expression and localization in the carotid wire injury mouse model. Immunofluorescence analysis revealed that TTK was colocalized with VSMCs but not with endothelial cells. The percentage of TTK‐positive, tdTomato‐positive VSMCs in the neointima increased progressively on days 14 and 28 postinjury (Figures 1E,F and S1C, Supporting Information). Meanwhile, the neointima area was linearly and positively correlated with the percentage of TTK‐positive, tdTomato‐positive VSMCs in neointima (Figure 1G). Consistently, quantitative real‐time polymerase chain reaction (qRT‐PCR) and western blot analyses revealed that the TTK mRNA and protein levels were gradually upregulated in the injured arteries of the carotid wire injury mouse model when compared with that in the arteries of the control mice on days 7, 14, and 28 post‐surgery (Figure S1D,E, Supporting Information).

Neointima formation is a critical pathophysiological process during atherosclerosis.^[^ ^5^, ^20^, ^21^ ^]^ To examine the expression of TTK in this context, we analyzed mouse atherosclerotic plaques using SMC‐lineage tracing *ApoE^−/−^* mice fed a high‐fat diet (HFD). Immunofluorescence analysis revealed a substantial increase in the percentage of TTK‐positive, tdTomato‐positive VSMCs in atherosclerotic plaques compared with healthy aortic arteries (Figure 1H,I). Moreover, the plaque area showed a positive linear correlation with the percentage of TTK‐positive, tdTomato‐positive VSMCs (Figure 1J). Additionally, qRT‐PCR and western blot analyses demonstrated that TTK mRNA and protein levels were significantly upregulated in the atherosclerotic arteries of *ApoE^−/−^* mice compared with control arteries (Figure S1F,G, Supporting Information).

Furthermore, in human samples, immunofluorescence analysis showed that TTK, which was nearly undetectable in healthy arteries, was expressed in human carotid atheroma and colocalized with VSMCs (Figure S2A, Supporting Information). Western blot analysis confirmed that TTK protein levels were elevated in human carotid atheroma compared with healthy arteries (Figure S2B, Supporting Information). Additionally, analysis of independent human atherosclerotic GEO datasets confirmed that TTK mRNA levels were upregulated in atherosclerotic plaques relative to non‐atherosclerotic arteries and further increased in advanced plaques compared to early‐stage plaques (Figures S2C,D, Supporting Information). Negative correlations were observed between TTK mRNA levels and SMC contractile markers across two independent human datasets, further highlighting the role of TTK in promoting phenotypic switching during atherosclerosis progression (Figure S2E–J, Supporting Information).

### 2.2 IRF1 Upregulates TTK Transcription in VSMCs upon Pathological Stimulation

**Figure 2:**
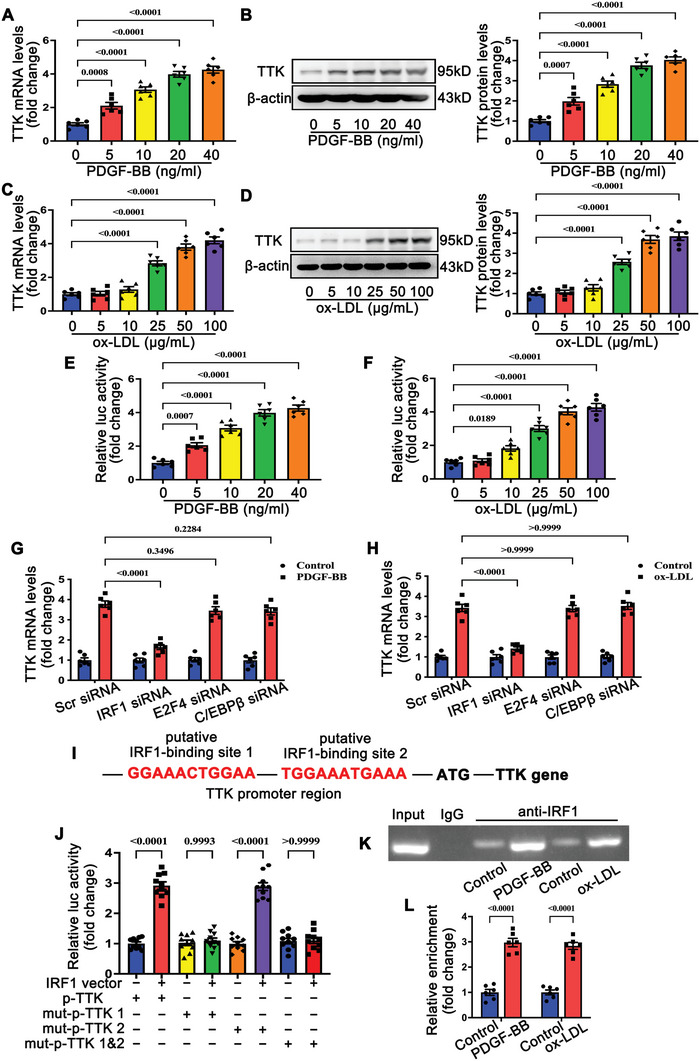
IRF1 upregulates TTK transcription in VSMCs upon pathological stimulation. A,B) Relative levels of TTK mRNA A) and protein B) in VSMCs stimulated with platelet‐derived growth factor‐BB (PDGF‐BB) (0, 5, 10, 20, and 40 ng mL^−1^) for 24 h (*n* = 6). C,D) Relative levels of TTK mRNA C) and protein D) in VSMCs stimulated with oxidized low‐density lipoprotein (ox‐LDL) (0, 5, 10, 25, 50, and 100 µg mL^−1^) for 24 h (*n* = 6). E) TTK promoter activity in MOVAS cells treated with PDGF‐BB (0, 5, 10, 20, and 40 ng mL^−1^) for 24 h (*n* = 6). F) TTK promoter activity in MOVAS cells treated with ox‐LDL (0, 5, 10, 25, 50, and 100 µg mL^−1^) for 24 h (*n* = 6). G) qRT‐PCR analysis of the relative mRNA level of TTK in VSMCs transfected with scrambled, IRF1, E2F4, or C/EBPβ‐specific siRNAs in the presence or absence of 20 ng mL^−1^ PDGF‐BB (*n* = 6). H) qRT‐PCR analysis of the relative mRNA level of TTK in VSMCs transfected with scrambled, IRF1, E2F4, or C/EBPβ‐specific siRNAs in the presence or absence of 20 ng mL^−1^ ox‐LDL (*n* = 6). I) Schematic illustration of putative IRF1 binding sequences in the TTK promoter region. J) Luciferase reporters of TTK promoter with native (p‐TTK), mutated IRF1binding site 1 (mut‐p‐TTK 1), mutated IRF1binding site 2 (mut‐p‐TTK 2), or mutated IRF1binding site 1 and 2 (mut‐p‐TTK 1&2) were cloned and co‐transfected with IRF1overexpressing vector into MOVAS cells for 48 h (*n* = 10). K,L) Chromatin immunoprecipitation (ChIP) assay K) and quantification L) demonstrated that PDGF‐BB and ox‐LDL promoted the binding of IRF1 to the TTK promoter (*n* = 6). IgG lane: negative control. Data are presented as the mean ± SEM; unpaired *t*‐test, one‐way ANOVA, two‐way ANOVA.

To elucidate the mechanism underlying TTK upregulation in postinjury neointimal formation and atherosclerosis, we examined its regulation in VSMCs under pathological conditions. Platelet‐derived growth factor BB (PDGF‐BB) and oxidized low‐density lipoprotein (ox‐LDL) are both known in vitro stimulators that mimic the pathological environment of postinjury restenosis and atherosclerosis.^[^ ^6^ ^]^ Treatment with PDGF‐BB or ox‐LDL resulted in a dose‐dependent increase in TTK mRNA and protein levels in VSMCs (**Figure** **2**A‐D), indicating transcriptional regulation of TTK under these conditions. Luciferase reporter assays further confirmed the transcriptional activation of the TTK promoter (Figure 2E,F). Next, the potential transcription factors regulating TTK expression were identified. Three transcription factors (IRF1, E2F4, and C/EBPβ) were predicted to potentially regulate TTK transcription using the Signaling Pathways Project database (www.signalingpathways.org), JASPAR software (http://jaspar.genereg.net) and literature review.^[^ ^12^ ^]^ Specific small interfering RNA (siRNA)‐mediated knockdown experiments revealed that only IRF1 silencing suppressed the PDGF‐BB‐ or ox‐LDL‐induced upregulation of TTK mRNA levels (Figures 2G,H and S3A–C, Supporting Information). In contrast, IRF1 overexpression significantly increased TTK expression (Figure S3D, Supporting Information). Two potential IRF1 binding sites were predicted within the TTK promoter region (Figure 2I). Luciferase reporter assays showed that mutation of binding site 1, but not binding site 2, abolished IRF1‐induced activation of the TTK promoter (Figure 2J). Chromatin immunoprecipitation (ChIP) assays confirmed that IRF1 interacts with the TTK promoter at binding site 1, and this interaction was enhanced following PDGF‐BB or ox‐LDL stimulation (Figure 2K,L). Additionally, the GEO dataset GSE43292 analysis revealed that IRF1 expression is elevated in human carotid atheroma and positively correlates with TTK expression in the human carotid artery (Figures S3E,F, Supporting Information). Consistently, ChIP assays demonstrated increased interaction between IRF1 and the TTK promoter during postinjury neointimal formation and atherosclerosis (Figure S3G,H, Supporting Information). These results indicated that IRF1 upregulates the transcription of TTK in VSMCs upon pathological stimulation.

### 2.3 TTK Knockout in VSMC Inhibits Postinjury Neointima Formation and Atherosclerosis In Vivo

**Figure 3:**
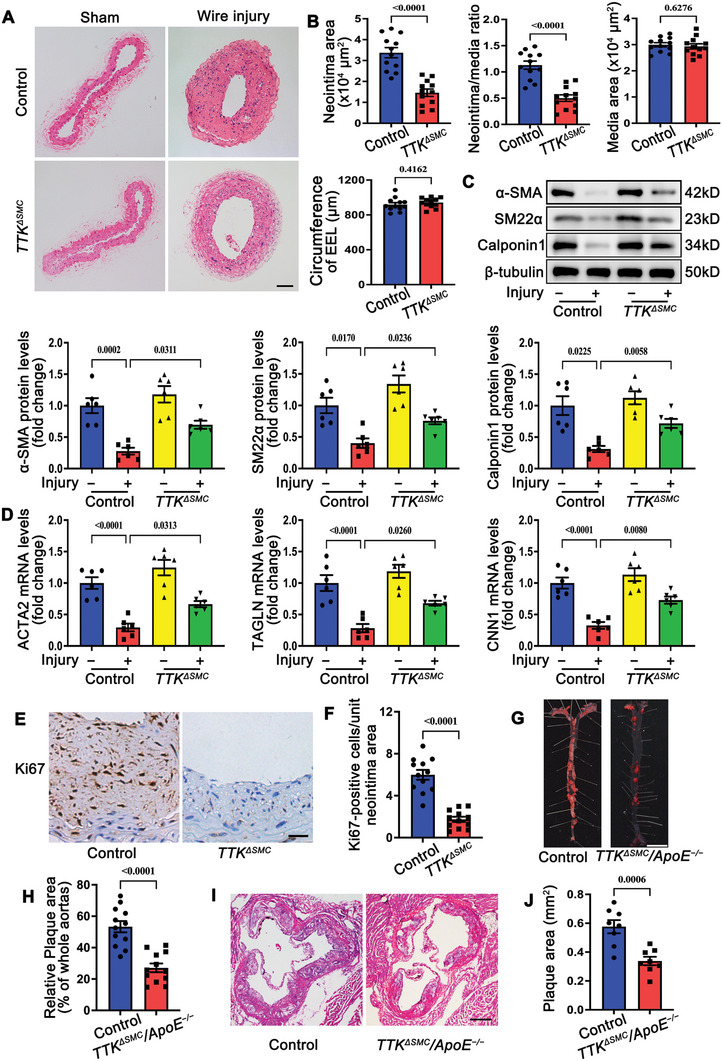
Smooth muscle cell (SMC)‐specific TTK deficiency suppresses neointima and plaque formation in vivo. A) Representative hematoxylin and eosin (HE)‐stained sections of sham‐operated and wire‐injured carotid arteries of control and *Ttk^ΔSMC^* mice on day 28 post‐surgery. Scale bar = 50 µm. B) Quantitative analysis of the neointima area, neointima‐to‐media ratio, media area, and external elastic lamina (EEL) circumference in the histological sections of wire‐injured carotid arteries (*n* = 12). C) Representative western blotting and quantification of α‐SMA, SM22α, and calponin1 in carotid arteries of control and *Ttk^ΔSMC^* mice on day 28 post‐sham or wire injury operations (*n* = 6). D) Relative mRNA levels of ACTA2, TAGLN, and CNN1 in carotid arteries of control and *Ttk^ΔSMC^* mice on day 28 post‐sham or wire injury operations (*n* = 6). E,F) Representative images of Ki67 immunohistochemical staining E) and corresponding quantification of Ki67‐positive cells in the neointima F) in the wire‐injured carotid artery sections of control and *Ttk^ΔSMC^* mice on day 28 post‐surgery (*n* = 12). Scale bar = 20 µm. G) Representative images of Oil Red O‐stained sections of whole aortas of control and *Ttk^ΔSMC^*/*ApoE^−/−^* mice fed on a high‐fat diet (HFD) for 16 weeks. Scale bar = 5 mm. H) Quantification of the plaque area in the whole aorta (*n* = 12). I) Representative HE‐stained sections of aortic root from control and *Ttk^ΔSMC^*/*ApoE^−/−^* mice fed on HFD for 16 weeks. Scale bar = 250 µm. J) Quantification of the plaque area in the aortic sinuses (*n* = 8). Data are presented as the mean ± SEM; unpaired *t*‐test, one‐way ANOVA.

To investigate the in vivo role of TTK in postinjury neointima formation and atherosclerosis, tamoxifen‐inducible SMC‐specific TTK knockout mice were generated by crossing *Ttk^fl^*/^fl^ mice with Myh11‐CreERT2 mice (Figure S4A,B, Supporting Information). Tamoxifen administration successfully induced Ttk deletion in SMCs of Ttk^fl/fl^/Myh11‐CreERT2 (Ttk^ΔSMC^) mice (Figure S4C,D, Supporting Information). The knockout did not affect body weight or blood pressure (Figure S4E, Supporting Information). Baseline histological analysis of contralateral uninjured carotid arteries via hematoxylin and eosin (HE) staining revealed no significant morphological differences between Ttk^ΔSMC^ mice and their control littermates (**Figure** **3**A). However, in injured carotid arteries, Ttk^ΔSMC^ mice displayed impaired neointima formation on day 28 post‐carotid wire injury. The neointima area and the neointima‐to‐media area ratio were significantly reduced in Ttk^ΔSMC^ mice compared to controls, while the media area and the external elastic lamina (EEL) circumference remained unaffected (Figure 3B). Furthermore, qRT‐PCR and western blot analyses showed that SMC‐specific Ttk deletion restored the expression of VSMC contractile markers that were suppressed by vascular injury (Figure 3C,D). Immunohistochemical analysis of Ki67 revealed a significant reduction in the number of Ki67‐positive proliferative cells in the neointima of Ttk^ΔSMC^ mice compared to controls (Figure 3E,F). Similarly, in a carotid ligation injury model, Ttk^ΔSMC^ mice exhibited attenuated neointima hyperplasia and reduced Ki67‐positive cells on day 28 post‐surgery (Figure S4F–I, Supporting Information).

To assess the role of TTK in atherosclerosis, Ttk^ΔSMC^ mice were crossed with ApoE*^−/−^* mice to obtain *Ttk^fl/^* ^fl^/Myh11‐CreERT2/ApoE*^−/−^* (Ttk^ΔSMC^/ApoE*^−/−^*) mice. There were no significant differences in body weight, blood pressure or serum cholesterol levels between knockout and control mice fed an HFD (Figure S5A–F, Supporting Information). However, en face Oil Red O staining demonstrated that SMC‐specific Ttk knockout reduced the aortic atherosclerotic plaque area in HFD‐fed ApoE*^−/−^* mice (Figure 3G,H). Consistently, HE staining of aortic root cross‐sections corroborated the reduced atherosclerotic plaque area in Ttk^ΔSMC^/ApoE*^−/−^* mice (Figure 3I,J).

### 2.4 TTK Promotes the Phenotypic Switching of VSMC In Vitro

**Figure 4:**
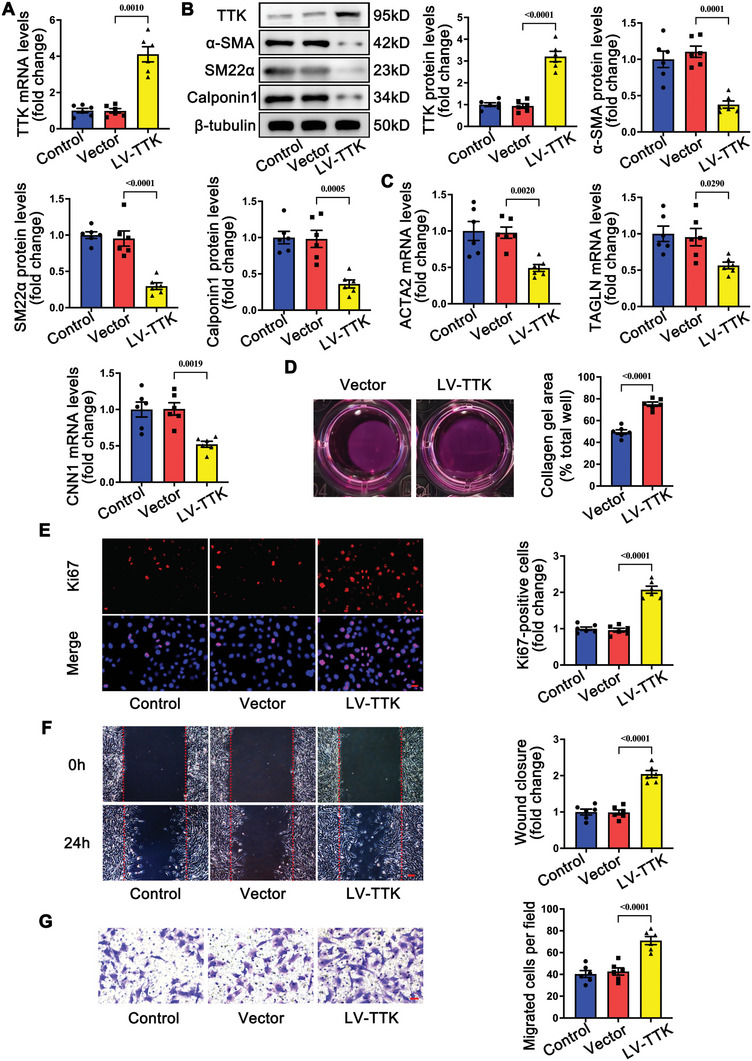
TTK overexpression promotes the phenotypic switching of VSMCs in vitro. A) VSMCs were transfected with empty vector or lentivirus encoding TTK overexpression construct (LV‐TTK). qRT‐PCR analysis of the relative mRNA level of TTK in VSMCs transfected with empty vector or LV‐TTK (*n* = 6). B) Representative western blotting and quantification of TTK, α‐SMA, SM22α, and calponin1 in VSMCs transfected with empty vector or LV‐TTK (*n* = 6). C) Relative mRNA levels of ACTA2, TAGLN, and CNN1 in VSMCs transfected with empty vector or LV‐TTK (*n* = 6). D), Representative images and quantification of collagen gel contraction containing VSMCs transfected with empty vector or LV‐TTK (*n* = 6). E) VSMCs were subjected to Ki67 immunohistochemical staining (red) and DAPI (blue) staining after transfection with empty vector or LV‐TTK. Representative immunofluorescence images and corresponding quantification of Ki67‐positive VSMCs are shown (*n* = 6). Scale bar = 20 µm. F,G) The migration ability of VSMCs transfected with empty vector or LV‐TTK was assessed using the wound healing F) and transwell assays G). Representative images and corresponding quantification of migration areas and migrated cells are shown (*n* = 6). Scale bar = 100 µm (upper) or 50 µm (lower). Data are presented as the mean ± SEM; unpaired *t*‐test, one‐way ANOVA.

To evaluate the impact of TTK on the phenotypic switching of VSMCs, TTK was overexpressed in mouse VSMCs using a lentivirus encoding TTK overexpression constructs (LV‐TTK). qRT‐PCR and western blot analyses confirmed a significant upregulation of TTK mRNA and protein levels in LV‐TTK‐transfected VSMCs compared to those transfected with an empty vector (**Figure** **4**A,B). TTK overexpression markedly suppressed the mRNA and protein levels of classical VSMC contractile phenotype markers (Figure 4B,C). Meanwhile, the collagen gel contraction assay indicated that LV‐TTK‐transfected VSMCs exhibited reduced contractility compared to empty vector‐transfected VSMCs (Figure 4D). Ki67 immunofluorescence staining demonstrated a significant increase in the proportion of proliferating (Ki67‐positive) VSMCs upon TTK overexpression (Figure 4E). Additionally, wound healing and Transwell assays revealed enhanced migratory capabilities in LV‐TTK‐transfected VSMCs compared to controls (Figure 4F,G).

Furthermore, specific siRNAs were used to silence TTK expression in VSMCs in vitro. The mRNA and protein levels of TTK were significantly downregulated upon transfection with two specific siRNAs (TTK siRNA‐1 and TTK siRNA‐2) (Figure S6A,B, Supporting Information). TTK knockdown promoted the expression of VSMC contractile markers and significantly enhanced VSMC contractility (Figure S6B,C Supporting Information). Moreover, Ki67 immunofluorescence analysis, wound healing, and Transwell assays demonstrated that TTK knockdown suppressed the proliferation and migration of VSMCs (Figure S6D–F, Supporting Information).

These findings suggest that TTK promotes the phenotypic switching of VSMCs in vitro whereas TTK knockdown reverses these effects.

### 2.5 TTK Induces VSMC Phenotypic Switching Through the Phosphorylation of p120‐Catenin at Threonine 310

**Figure 5:**
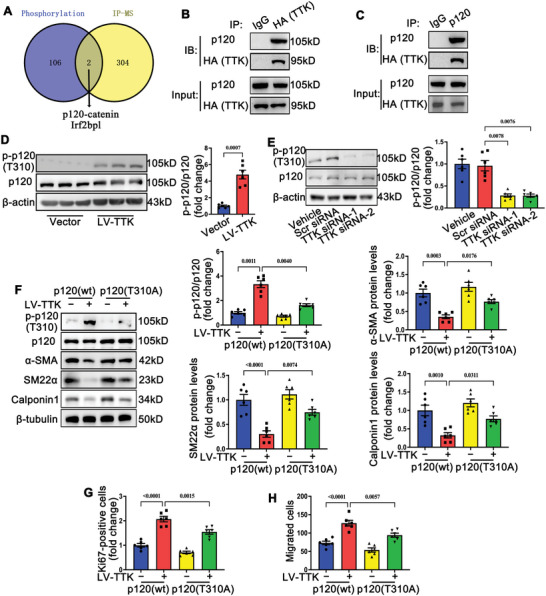
TTK promotes VSMCs phenotypic switching by phosphorylating p120‐catenin at T310. A) Overlapping analysis of the upregulating phosphorylation proteins in hemagglutinin (HA)‐tagged TTK construct‐transfected group in phosphorylated proteomics and interacting proteins of TTK. B) Lysates of VSMCs transfected with the hemagglutinin (HA)‐tagged TTK lentivirus were immunoprecipitated with anti‐HA antibodies, and the precipitates were analyzed using immunoblotting with anti‐p120 antibodies. C) Lysates of VSMCs transfected with the HA‐tagged TTK lentivirus were immunoprecipitated with anti‐p120 antibodies, and the precipitates were analyzed using immunoblotting with anti‐HA antibodies. D) Representative western blotting and quantification of p120 phosphorylated at T310 in VSMCs transfected with empty vector or LV‐TTK (*n* = 6). E) Representative western blotting and quantification of p120 phosphorylated at T310 in VSMCs transfected with scramble small interfering RNA (siRNA) or TTK‐specific siRNAs (*n* = 6). F) Representative western blotting and quantification of p120 phosphorylated at T310, α‐SMA, SM22α, and calponin1 in VSMCs co‐transfected with vector or LV‐TTK and wild‐type p120 (p120‐wt) or mutant p120 (p120‐T310A) (*n* = 6). G,H) Quantification of Ki67 immunofluorescence staining G) and transwell assay results H) of VSMCs co‐transfected with empty vector or LV‐TTK and p120‐wt or p120‐T310A vector (*n* = 6). Data are presented as the mean ± SEM; unpaired *t*‐test, one‐way ANOVA.

To elucidate the downstream molecular mechanisms underlying TTK‐mediated phenotypic switching in VSMCs, the study investigated TTK's role as a bispecific protein kinase. Hemagglutinin (HA)‐tagged TTK overexpression constructs or control vectors were transfected into VSMCs in vitro, followed by quantitative phosphoproteomic analysis to identify TTK‐dependent phosphorylation events (Data S2, Supporting Information). This analysis identified 153 upregulated and 783 downregulated phosphorylation sites in the HA‐tagged TTK‐overexpressing group compared to the control group (Figure S7A,B, Supporting Information). Mass spectrometry analysis of proteins pulled down by anti‐HA antibodies identified potential TTK‐interacting proteins (Data S3, Supporting Information). Overlapping these interacting proteins with upregulated phosphoproteins revealed that p120‐catenin (p120) and Irf2bpl might mediate TTK's downstream effects in VSMCs (**Figure** **5**A). Co‐immunoprecipitation assays confirmed that TTK interacted with p120‐catenin but not with Irf2bpl (Figures 5B,C and S7C, Supporting Information). Phosphoproteomic data indicated that TTK overexpression enhanced the phosphorylation of p120‐catenin at threonine 310 (T310), suggesting that TTK may phosphorylate p120‐catenin at T310 to mediate phenotypic switching in VSMCs.

Further validation showed that TTK overexpression increased p120 phosphorylation at T310, while TTK knockdown inhibited it (Figure 5D,E). To assess the functional role of T310 phosphorylation, a mutant construct of p120‐catenin with T310 substituted by alanine (p120‐T310A) was generated, along with the wild‐type p120 (p120‐wt) construct (Figure S7D, Supporting Information). Transfection of VSMCs with p120‐T310A significantly impaired TTK‐induced p120 phosphorylation at T310 (Figure 5F). Compared to p120‐wt, p120‐T310A inhibited TTK‐induced VSMC phenotypic switching. This was evidenced by increased expression of VSMC contractile markers and reduced VSMC proliferation and migration (Figures 5F–H and S7E–G, Supporting Information). Thus, the phosphorylation p120 at T310 is a critical mechanism mediating the TTK‐induced phenotype switching of VSMCs.

**Figure 6:**
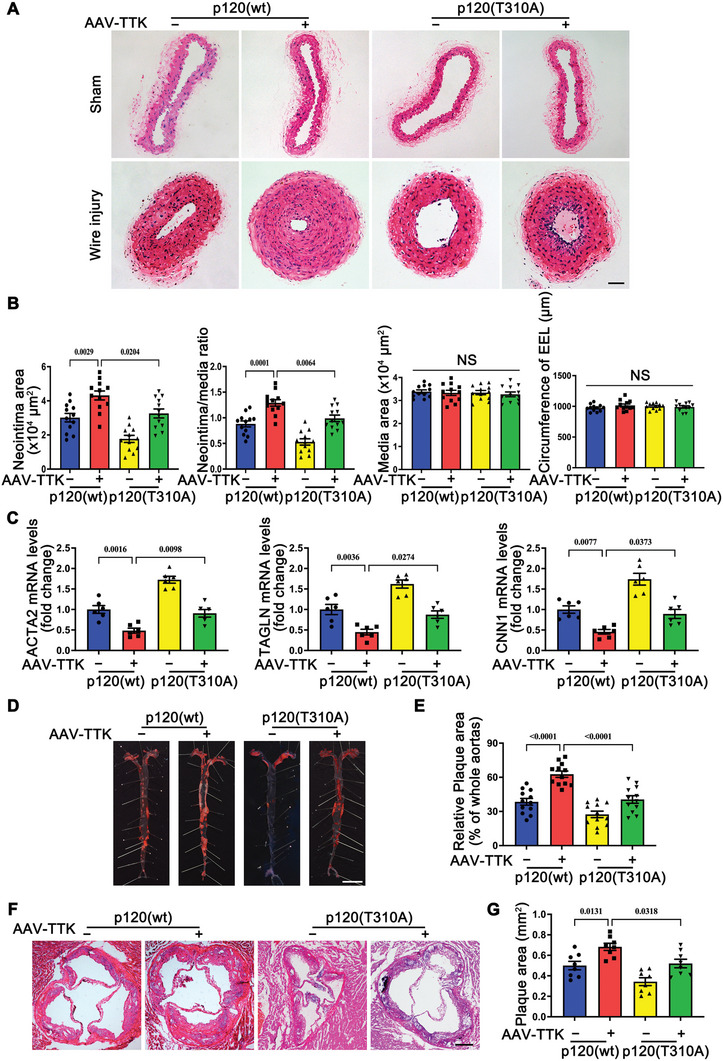
TTK induces postinjury neointima formation and atherosclerosis through the phosphorylation of p120‐catenin at T310 in vivo. A–C) *Myh11‐CreER^T2^* mice were intraperitoneally injected with tamoxifen for five consecutive days to induce Cre expression. On day 7 post‐final injection, the mice were intravenously injected with different virus. Carotid artery wire injury was induced on day 10 post‐virus injection. The mice were euthanized, and the carotid arteries were harvested on day 14 post‐surgery. A) Representative hematoxylin and eosin (HE)‐stained carotid sections showing the neointima on day 14 post‐carotid artery wire injury. Scale bar = 50 µm. B) Quantitative analysis of the neointima area, neointima‐to‐media ratio, media area, and EEL circumference in carotid sections of different groups (*n* = 12). C) Relative mRNA levels of ACTA2, TAGLN, and CNN1 in carotid arteries of different groups (*n* = 6). D–G) *Myh11‐CreER^T2^*/*ApoE^−/−^* mice were intraperitoneally injected with tamoxifen for five consecutive days to induce Cre expression. On day 7 post‐final injection, the mice were intravenously injected with different virus. High‐fat diet (HFD) was fed on day 10 post‐virus injection. The mice were euthanized at week 12 post‐HFD feeding for subsequent experiments. D,E) Representative image and quantification of the plaque area in Oil Red O‐stained whole aortas (*n* = 12). F,G) Representative hematoxylin and eosin (HE) staining and quantification of the plaque area in aortic root sections. Scale bar = 250 µm (*n* = 8). Data are presented as the mean ± SEM; one‐way ANOVA. NS, non‐significant.

The involvement of the TTK/phosphorylated p120‐catenin (T310) pathway in vivo was examined in vascular injury and atherosclerosis models. Phosphorylation of p120 at T310 was significantly upregulated in the neointima of vascular injury mouse models (Figure S8A, Supporting Information). SMC‐specific TTK deletion notably suppressed p120 T310 phosphorylation during vascular injury in vivo (Figure S8B, Supporting Information). SMC‐specific TTK overexpression increased p120 T310 phosphorylation in carotid and aortic arteries transfected with p120‐wt but not in those transfected with the phosphorylation‐defective p120‐T310A mutant, under wire injury, or HFD‐fed *ApoE^−/−^* mouse conditions (Figure S8C‐E, Supporting Information). HE staining revealed that SMC‐specific TTK overexpression exacerbated wire injury‐induced neointima hyperplasia (**Figure** **6**A). However, transfection of p120‐T310A into injured carotid arteries significantly attenuated TTK‐induced neointima formation (Figure 6A,B) and reversed the inhibitory effects of TTK on VSMC contractile marker expression postinjury (Figure 6C). Similarly, en face Oil Red O staining demonstrated that SMC‐specific TTK overexpression significantly increased aortic atherosclerotic plaque formation in arteries transfected with p120‐wt but not in those transfected with p120‐T310A, under HFD‐fed *ApoE^−/−^* mouse conditions (Figures 6D,E). HE staining of aortic roots revealed consistent changes in plaque area, further supporting the role of p120 phosphorylation at T310 in TTK‐mediated effects (Figure 6F,G).

These findings underscore that TTK promotes postinjury neointima formation and atherosclerosis in vivo by driving the phenotypic switching of VSMCs through the phosphorylation of p120‐catenin at T310.

### 2.6 TTK‐Induced VSMC Phenotypic Switching is Dependent on β‐Catenin Nuclear Accumulation and the Subsequent MYOCD/SRF Complex Dissociation

**Figure 7:**
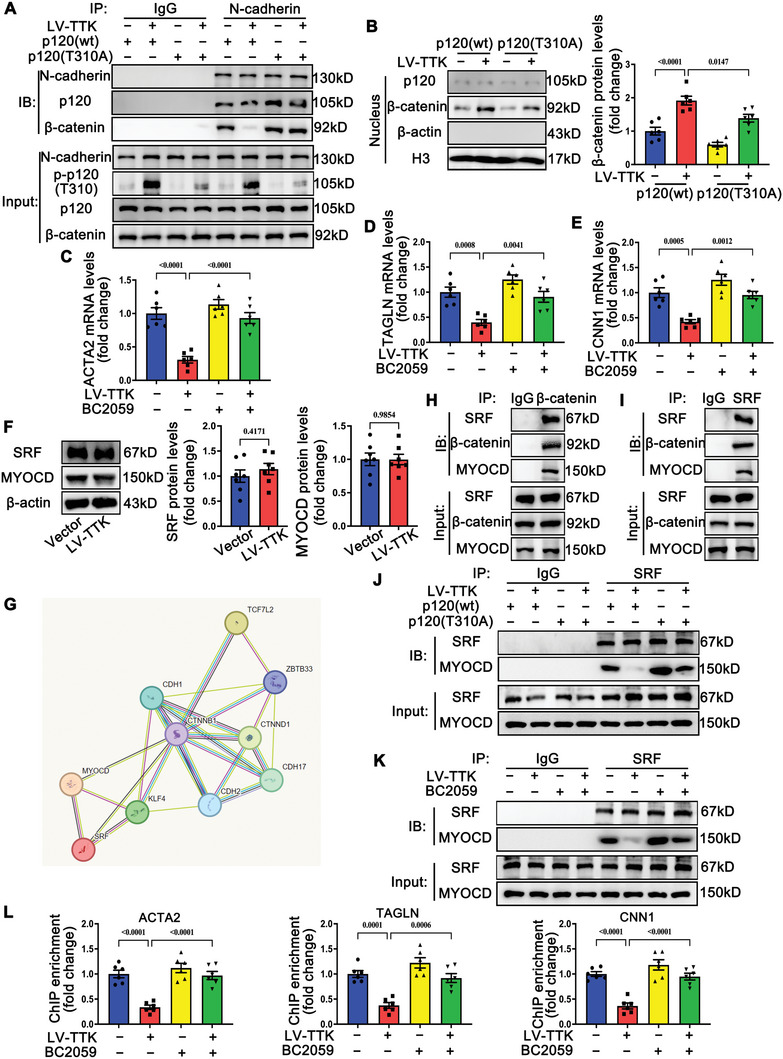
TTK‐induced phenotypic switching of VSMCs is dependent on β‐catenin nuclear accumulation and the subsequent myocardin/SRF complex dissociation. A) VSMCs were co‐transfected with empty vector or lentivirus encoding TTK overexpression construct (LV‐TTK and wild‐type p120 (p120‐wt) or mutant p120 (p120‐T310A) constructs. The cell lysates were immunoprecipitated with anti‐N‐cadherin antibodies. B) Representative western blotting and quantification of nuclear p120 and β‐catenin protein levels in VSMCs co‐transfected with empty vector or LV‐TTK and p120‐wt or p120‐T310A. Protein levels were normalized to H3 levels (*n* = 6). C–E) Relative mRNA levels of ACTA2 C), TAGLN D), and CNN1 E) in VSMCs transfected with vector or LV‐TTK and treated with or without 40 nm BC2059 (*n* = 6). F) Representative western blotting and quantification of SRF and MYOCD in VSMCs transfected with empty vector or LV‐TTK (*n* = 6). G) STRING database predicted that CTNNB1 (β‐catenin) interacts with MYOCD and SRF. H) Lysates of VSMCs were immunoprecipitated with anti‐β‐catenin antibodies. I)Lysates of VSMCs were immunoprecipitated with anti‐SRF antibodies. J) VSMCs were co‐transfected with empty vector or LV‐TTK and p120‐wt or p120‐T310A. The cell lysates were immunoprecipitated with anti‐SRF antibodies. K) VSMCs were transfected with empty vector or LV‐TTK in the presence or absence of 40 nm BC2059. The cell lysates were immunoprecipitated with anti‐SRF antibodies. L) ChIP analyzing the binding of SRF to promoters of ACTA2, TAGLN, and CNN1 in VSMCs transfected with vector or LV‐TTK and treated with or without 40 nm BC2059 (*n* = 6). Data are presented as the mean ± SEM; unpaired *t*‐test, one‐way ANOVA.

The downstream mechanisms of the TTK‐phosphorylated p120 (T310) pathway were further investigated. Previous studies have shown that p120‐catenin forms a complex with N‐cadherin and β‐catenin at the membrane to facilitate cell‐cell adhesion.^[^ ^22^, ^23^ ^]^ This study explored how TTK‐mediated phosphorylation of p120 influences this complex in VSMCs. Overexpression of TTK reduced β‐catenin binding to N‐cadherin and promoted β‐catenin dissociation from the N‐cadherin/p120/β‐catenin complex in VSMCs, whereas the p120‐T310A mutant inhibited this dissociation (**Figure** **7**A). In contrast, the p120‐T310A mutant significantly inhibited β‐catenin dissociation from the complex (Figure 7A). Previous studies report that β‐catenin dissociated from cancer cell membranes translocate from the cytoplasm to the nucleus, wherein it activates transcriptional factors and consequently, promotes the metastatic potential of cancer cells.^[^ ^24^ ^]^ We hypothesized that TTK‐induced VSMC phenotypic switching involves β‐catenin nuclear accumulation. Analysis of nuclear extracts confirmed that TTK overexpression significantly increased nuclear β‐catenin levels, an effect that was suppressed by the p120‐T310A mutant (Figure 7B). Then, tegatrabetan (BC2059, a β‐catenin antagonist) was used to suppress β‐catenin nuclear translocation (Figure S9A, Supporting Information). BC2059 significantly suppressed the effect of TTK on contractile marker expression and VSMC proliferation and migration (Figures 7C–E and S9B,C, Supporting Information). These findings indicate that β‐catenin nuclear accumulation mediates TTK‐induced VSMC phenotypic switching.

Further investigation focused on the role of β‐catenin in the regulation of the MYOCD/SRF pathway, a critical regulator of smooth muscle‐specific gene expression.^[^ ^25^, ^26^ ^]^ While TTK overexpression did not alter the protein levels of MYOCD or SRF in VSMCs (Figure 7F), STRING database (https://string‐db.org/) analysis predicted a potential interaction between β‐catenin (gene name: CTNNB1) and the MYOCD/SRF complex (Figure 7G). Co‐immunoprecipitation experiments validated the endogenous interaction of β‐catenin with the MYOCD/SRF complex in VSMCs (Figure 7H,I). Importantly, TTK overexpression promoted the dissociation of the MYOCD/SRF complex, an effect reversed by p120‐T310A mutant transfection or BC2059 treatment (Figure 7J,K). Furthermore, ChIP assays demonstrated that TTK overexpression reduced SRF occupancy on the promoters of ACTA2, TAGLN, and CNN1 genes in VSMCs, which was blocked by p120‐T310A mutant transfection or BC2059 treatment (Figures 7L and S9D, Supporting Information).

Additionally, SMC‐specific *Ttk* knockout mice subjected to vascular injury were used to verify these mechanisms in vivo. SMC‐specific *Ttk* knockout significantly inhibited β‐catenin dissociation from the N‐cadherin/p120/β‐catenin complex, β‐catenin nuclear translocation, and MYOCD/SRF complex dissociation in the injured carotid arteries (Figure S10A–C, Supporting Information).

These results collectively demonstrate that TTK promotes VSMC phenotypic switching by inducing p120‐catenin (T310) phosphorylation, enhancing β‐catenin nuclear accumulation, and driving MYOCD/SRF complex dissociation.

### 2.7 TTK Inhibitor Effectively Suppresses Postinjury Neointima Formation and Atherosclerosis In Vivo

**Figure 8:**
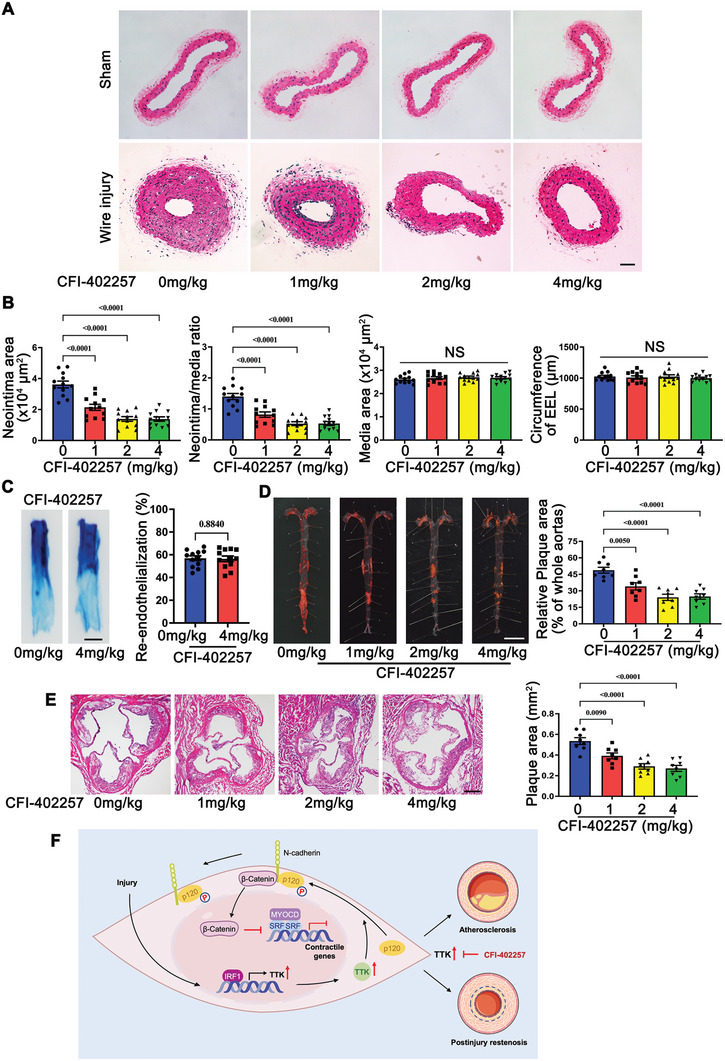
Effects of TTK inhibitor on postinjury neointima formation and atherosclerosis in vivo. A) Representative HE‐stained sections of sham‐operated and wire‐injured carotid arteries from C57 mice administered with CFI‐402257 (1, 2, or 4 mg kg^−1^ body weight) on day 28 post‐surgery. Scale bar = 50 µm. B) Quantitative analysis of the neointima area, neointima‐to‐media ratio, media area, and EEL circumference in the histological sections of wire‐injured carotid arteries from C57 mice administered with CFI‐402257 on day 28 post‐surgery (*n* = 12). C) Representative images of en face Evans blue‐stained carotid arteries from the C57 mice administered with vehicle or CFI‐402257 (4 mg kg^−1^ body weight) at 5 days after wire injuries. Scale bar = 1 mm. Quantification of re‐endothelialization in the injured carotid arteries (*n* = 12). D) Representative images of en face Oil Red O‐stained whole aorta sections from *ApoE^−/−^* mice administered with CFI‐402257 (1, 2, or 4 mg kg^−1^ body weight) and fed on a high‐fat diet (HFD) for 12 weeks. Scale bar = 5 mm. Quantification of the plaque area in the whole aorta (*n* = 8). E) Representative HE‐stained aortic root sections from *ApoE^−/−^* mice administered with CFI‐402257 and fed on an HFD for 12 weeks. Scale bar = 250 µm. Quantification of the plaque area in the aortic root sections (*n* = 8). F) Schematic illustration of TTK‐induced phenotypic switching of VSMCs to promote postinjury neointima formation and atherosclerosis. Data are presented as the mean ± SEM; unpaired *t*‐test, one‐way ANOVA. NS, non‐significant.

The results of this study suggest that aberrant upregulation of TTK contributes to the phenotypic switching of VSMCs, leading to both postinjury neointima formation and atherosclerosis. To explore the therapeutic potential of targeting TTK, the study evaluated the effects of CFI‐402257, a highly specific, selective, and potent TTK inhibitor, in preventing postinjury neointima formation and treating atherosclerosis. In vitro, CFI‐402257 dose‐dependently induced the expression of contractile markers and inhibited VSMC proliferation and migration (Figure S11A–E, Supporting Information). Moreover, CFI‐402257 effectively blocked the effects of TTK overexpression on p120 phosphorylation at T310, as well as on VSMC contractile markers, proliferation, and migration (Figure S11F–K, Supporting Information). Additionally, the therapeutic potential of CFI‐402257 in vivo was examined using the carotid wire injury mouse model and HFD‐fed *ApoE^−/−^* mice. Notably, oral administration of CFI‐402257 significantly inhibited neointimal formation and reduced the intima/media ratio in the carotid wire injury model, compared with vehicle control, without affecting the media area or the circumference of the EEL (**Figure** **8**A,B). Notably, CFI‐402257 did not impair reendothelialization following carotid wire injury (Figure 8C). Additionally, the safety of CFI‐402257 administration was evaluated in vivo. HE staining showed no pathological changes in the brain, heart, liver, spleen, kidney, or lung tissues after treatment (Figure S12A, Supporting Information). Meanwhile, there were no significant differences in body weight between the treatment groups (Figure S12B, Supporting Information). Blood markers, including urea nitrogen, creatinine, aspartate transaminase, and alanine transaminase, indicated that CFI‐402257 did not induce hepatotoxicity or nephrotoxicity (Figure S12C, Supporting Information).

In HFD‐fed *ApoE^−/−^* mice, en face Oil Red O staining demonstrated that CFI‐402257 administration reduced the aortic atherosclerotic plaque area (Figure 8D), and HE staining of aortic root cross‐sections revealed similar reductions in the atherosclerotic plaque area (Figure 8E). Importantly, there were no significant changes in body weight, blood lipid levels, or kidney and liver functions between the treatment groups (Figure S12D–F, Supporting Information).

## 3 Discussion

Despite the widespread use of lipid‐lowering drugs, these treatments address only about one‐third of the overall cardio‐cerebrovascular risk. Moreover, vascular restenosis and thrombotic events following surgical interventions continue to contribute significantly to treatment failure.^[^ ^3^, ^4^ ^]^ This underscores the urgent need for novel therapeutic strategies to manage atherosclerosis, prevent postinjury restenosis, and mitigate thrombotic complications. This study identifies TTK as a novel regulator of VSMC phenotypic switching, a critical process in the progression of postinjury neointimal formation and atherosclerosis. In both carotid injury and atherosclerotic mouse models, TTK expression was progressively upregulated as the disease advanced. Functionally, TTK facilitated the phenotypic switching of VSMCs by downregulating contractile phenotype markers and enhancing proliferation and migration capabilities. Mechanistically, TTK directly phosphorylated p120‐catenin at T310, leading to β‐catenin nuclear accumulation and subsequent dissociation of the MYOCD/SRF complex. Furthermore, SMC‐specific knockout of TTK inhibited postinjury neointimal formation in mouse models of carotid wire and ligation‐induced injury, as well as reduced atherosclerotic lesions in *ApoE^−/−^* mice. Furthermore, oral administration of the TTK inhibitor CFI‐402257 demonstrated significant therapeutic efficacy by reducing neointimal formation and atherosclerotic lesion size in these models. Notably, CFI‐402257 did not impair reendothelialization after carotid wire injury (Figure 8F). This study is the first to demonstrate the dual therapeutic potential of targeting TTK for treating atherosclerosis and preventing postinjury restenosis without adversely affecting endothelial repair.

TTK, a dual‐specific protein kinase, can phosphorylate tyrosine, serine, and threonine.^[^ ^12^ ^]^ Recent research has implicated TTK in tumorigenesis and the metastatic progression of several cancer types, where it contributes to poor clinical outcomes through diverse mechanisms.^[^ ^18^, ^19^ ^]^ Our study revealed that TTK promoted the phenotypic switching of VSMCs. Hence, to elucidate the downstream mechanisms, quantitative phosphoproteomic analysis was conducted on VSMCs overexpressing HA‐tagged. Previous studies indicated that TTK may directly or indirectly bind and phosphorylate its downstream substrate to perform its function.^[^ ^27^ ^]^ Co‐immunoprecipitation combined with mass spectrometry identified p120‐catenin as a potential downstream substrate of TTK. TTK was shown to phosphorylate p120‐catenin at T310, and this phosphorylation was critical for TTK‐induced VSMC phenotypic switching. The phosphorylation‐defective p120 mutant (p120‐T310A) significantly attenuated the effects of TTK on VSMC phenotype switching in vitro. Furthermore, in vivo studies demonstrated that TTK overexpression exacerbated wire injury‐induced neointimal formation and atherosclerotic lesions in *ApoE* ^***−/−***^ mice transfected with p120‐wt constructs, while these effects were markedly reduced in mice transfected with p120‐T310A constructs. These findings establish that TTK promotes VSMC phenotypic switching through phosphorylation of p120‐catenin at T310, driving postinjury neointimal formation and atherosclerosis. However, whether additional phosphorylation sites or alternative signaling pathways contribute to TTK‐mediated effects requires further study.

P120‐catenin forms complexes with N‐cadherin and β‐catenin in the membrane to mediate cell‐cell adhesion.^[^ ^22^ ^]^ The N‐cadherin/p120/β‐catenin complex dissociates under pathological conditions.^[^ ^28^ ^]^ This study demonstrated that TTK overexpression decreased the binding ability of β‐catenin to N‐cadherin and induced the nuclear accumulation of β‐catenin. Transcription factor SRF and its coactivator MYOCD are master factors regulating smooth muscle‐specific gene expression.^[^ ^25^, ^26^ ^]^ Protein interaction analysis and co‐immunoprecipitation verification revealed that β‐catenin interacts with the MYOCD/SRF complex. TTK overexpression promoted the dissociation of the MYOCD/SRF complex and reduced SRF occupancies on the promoters of smooth muscle‐specific genes, which was blocked upon p120‐T310A mutant transfection or BC2059 treatment. These results suggested that TTK promoted the phenotypic switching of VSMCs by upregulating β‐catenin nuclear accumulation and MYOCD/SRF complex dissociation. Future studies should determine if β‐catenin directly or indirectly interacts with the MYOCD/SRF complex. Previous studies have reported that nuclear β‐catenin could bind and activate transcriptional factors, enhancing the metastatic potential of cancer cells.^[^ ^29^, ^30^ ^]^ Whether TTK‐induced β‐catenin nuclear accumulation regulates phenotypic switching of VSMCs through other pathways needs to be further examined.

The in vivo effects of TTK in promoting postinjury neointimal formation and atherosclerosis highlight its translational potential. Several small‐molecule inhibitors targeting TTK have been developed and characterized for cancer treatment.^[^ ^31^, ^32^, ^33^ ^]^ Among these, three inhibitors have undergone clinical trials, with CFI‐402257 currently in phase 2 clinical trials (NCT05251714). This study selected CFI‐402257 to evaluate the therapeutic potential of TTK inhibitors in simultaneously preventing postinjury restenosis and treating atherosclerosis. In the carotid wire injury mouse model, postinjury neointimal formation was significantly reduced in the CFI‐402257‐treated group compared to the control group. Physiological, histological, and blood biochemical analyses indicated that CFI‐402257 administration did not induce systemic toxicity in vivo. Moreover, oral administration of CFI‐402257 inhibited atherosclerotic lesions in *ApoE* ^***−/−***^ mice without altering lipid levels. Notably, CFI‐402257 had no inhibitory effect on reendothelialization after carotid wire injury—a critical finding for the development of novel drug‐eluting stents. Current drug‐eluting stents used in clinical settings inhibit both VSMC phenotypic switching and reendothelialization, leading to increased rates of lumen thrombotic events.^[^ ^9^, ^34^ ^]^ These findings indicate that the TTK inhibitor CFI‐402257 represents a promising strategy to simultaneously prevent postinjury restenosis and lumen thrombotic events, as well as treat atherosclerosis. However, the concentration range of CFI‐402257 tested in this study was limited. Future investigations should explore a broader range of drug concentrations to identify the optimal dosage that maximizes therapeutic efficacy while minimizing adverse effects. Furthermore, while this study evaluated general toxicity, more comprehensive assessments of potential side effects, such as gastrointestinal disturbances and hematologic toxicities (e.g., myelosuppression), are necessary.

Although TTK overexpression has been reported in various cancers,^[^ ^15^, ^16^, ^17^ ^]^ its regulatory mechanisms remain poorly understood. This study demonstrated that TTK is upregulated in VSMCs in multiple disease models in vivo and in vitro following treatment with PDGF‐BB and ox‐LDL. Previous studies suggested that E2F4 inhibits TTK transcription.^[^ ^12^ ^]^ However, this study demonstrated that E2F4 is not involved in regulating expression. Instead, bioinformatics analysis and experimental validation identified IRF1 as a transcription factor promoting TTK expression. Notably, IRF1 has been implicated in atherosclerosis through various pathways.^[^ ^35^ ^]^ This study demonstrated that IRF1‐induced TTK expression partially mediates its pro‐atherosclerotic effects. Nevertheless, the mechanisms underlying IRF1 activation during neointimal formation remain to be elucidated in future research.

Recent studies have reported that N‐cadherin‐targeted melanin nanoparticles and photothermal treatment are promising strategies to regulate phenotypic switching of endothelial and cancer cells.^[^ ^36^, ^37^, ^38^ ^]^ Additionally, zeolitic imidazolate framework‐8 nanoparticles have been reported to directly alter VSMC actin organization and contractility.^[^ ^39^ ^]^ Future studies should explore whether these novel nanoparticles and photothermal treatments could similarly regulate VSMC phenotypic switching to prevent postinjury restenosis and treat atherosclerosis.

There are several limitations to this study. First, while atherosclerosis development involves multiple processes, including phenotypic switching of VSMCs, inflammation, oxidative stress, and endothelial dysfunction, this study primarily focused on the role of TTK in VSMC phenotypic switching. Further investigations are needed to determine whether TTK contributes to other atherosclerotic processes. Parameters such as inflammatory cell infiltration and elastic fiber degradation should be assessed to provide a more comprehensive understanding of TTK's role in atherosclerosis. Second, the use of virus‐derived p120‐T310A mutant vectors may partially interfere with experiments due to endogenous p120‐catenin. Future studies employing genetically engineered mice harboring p120‐T310A mutations are required to circumvent this limitation and provide more robust insights into the role of TTK and p120‐catenin in disease progression.

In conclusion, this study revealed that upregulated TTK promotes postinjury neointimal formation and atherosclerosis by facilitating VSMC phenotypic switching. This occurs through the direct phosphorylation of p120‐catenin at T310, leading to β‐catenin nuclear accumulation and dissociation of the MYOCD/SRF complex. Therefore, targeting TTK with specific inhibitors or alternative strategies presents a promising therapeutic approach to simultaneously prevent postinjury restenosis and manage atherosclerosis.

## 4 Experimental Section

### Human Tissue Samples

All protocols involving human tissue samples were approved by the Ethics Committee of Union Hospital, Tongji Medical College, Huazhong University of Science and Technology (Wuhan, China, 2020IEC‐J078) and strictly adhered to ethical standards. Informed consent was obtained from all patients participating in the study. Healthy artery samples, which were used as controls, were obtained from organ transplant donors. Human atherosclerotic samples were obtained from patients undergoing carotid endarterectomy. The clinical characteristics of patients are described in Table S1 (Supporting Information).

### Mice

All animal experiments were approved by the Institutional Animal Care and Use Committee of Tongji Medical College, Huazhong University of Science and Technology (Wuhan, China, TY20200175) and performed according to the Guide for the Care and Use of Laboratory Animals (NIH Publication, Eighth edition, 2011). *Myh11‐CreER^T2^* (stock number: 019079; Jackson Laboratory), *Rosa26^tdTomato^* (stock number: 007909; Jackson Laboratory), and *ApoE^−/−^* (stock number: 002052; Jackson Laboratory) mice were purchased from the Jackson Laboratory. *Ttk* floxed (*Ttk^fl/fl^*) mice (Strain NO. T012794) constructed with the clustered regularly interspaced palindrome repeats (CRISPR)/Cas9 system were purchased from GemPharmatech (Nanjing, China). Briefly, the fertilized eggs of C57BL/6JGpt mice were microinjected with the CRISPR/Cas9 construct and donor and transplanted to generate positive F0 mice. After validation using PCR and sequencing, positive F0 generation mice were mated with C57BL/6JGpt mice to obtain a stable F1 generation mouse model.

To generate the SMC lineage tracing murine model (*Myh11‐CreER^T2^*/*Rosa26^tdTomato^* mice), the *Myh11‐CreER^T2^* mice were bred with *Rosa26^tdTomato^* reporter mice. To generate these mice with an atheroprone background, *Myh11‐CreER^T2^*/*Rosa26^tdTomato^* mice were bred with *ApoE* ^−/−^ mice to obtain *Myh11‐CreER^T2^*/*Rosa26^tdTomato^*/*ApoE^−/−^* mice with further appropriate genotyping. *Ttk^fl/fl^* mice were crossed with *Myh11‐CreER^T2^* mice to generate experimental *Ttk^fl/fl^*/*Myh11‐CreER^T2^* (*Ttk^ΔSMC^*) mice and their littermate *Myh11‐CreER^T2^* (control) mice. *Ttk^ΔSMC^*/*ApoE^−/−^* mice and their littermate control *Myh11‐CreER^T2^*/*ApoE^−/−^* mice were then obtained by crossing *Ttk^ΔSMC^* and *ApoE* ^−/−^ mice. Experimental and control mice were age‐matched and size‐matched. Tamoxifen (ST1681, Beyotime) dissolved in corn oil (ST1177, Beyotime) (20 mg mL^−1^) was intraperitoneally injected into mice (75 mg kg^−1^ body weight) for five consecutive days to conditionally induce Cre recombinase expression, which resulted in tdTomato reporter gene expression and/or *Ttk* deletion. All mice were genotyped using PCR amplification of tail DNA samples with the specific primers listed in Table S2 (Supporting Information). Mice were maintained in a room under the following conditions: access to food and water, ad libitum; circadian cycle, 12‐h light‐dark cycle; humidity, 40–60%; temperature, 20–25 °C.

### Mouse Carotid Artery Ligation Model

Carotid artery ligation was performed in male mice aged 8–12 weeks (body weight: 20–25 g) as described previously.^[^ ^40^, ^41^ ^]^ Mice were completely anesthetized before surgery. The left common carotid artery was exposed through an anterior midline incision in the neck and separated from the vagus nerve and surrounding tissue. Next, the left common carotid artery was ligated proximal to the left carotid bifurcation with a 6‐0 silk braided non‐resorbable suture (SA82G, Ethicon) to block blood flow. Male mice in the sham control group were subjected to a similar surgery without ligation. After surgery, mice were euthanized on days 7, 14, and 28 post‐surgery. Phosphate‐buffered saline (PBS) was infused into the left ventricle to flush the remaining blood from the vessels. The carotid artery specimens were harvested for qRT‐PCR, western blotting, and histological analyses. For histological analyses, the carotid arteries were dissected and fixed for sections. Sections were obtained at 200 µm proximal to the ligature site on the common carotid according to previous study.^[^ ^42^ ^]^

### Mouse Carotid Artery Wire Injury Model

Carotid artery wire injury was induced in male mice aged 8–12 weeks by advancing a guide wire with a diameter of 0.015 inches (Cook, Bloomington, USA). After introducing a midline neck incision, the left common carotid artery was bluntly dissected. Temporary ligation of the left common carotid and internal carotid arteries was performed to interrupt blood flow during the procedure. The left external carotid artery was subsequently tied off distally, and an incision was introduced proximal to the ligature through which a guidewire was inserted ≈1 cm into the left common carotid artery. The wire was withdrawn to the carotid bifurcation with a rotating motion to denude the endothelium. This procedure was repeated three times before removing the wire. The left external carotid artery was then ligated proximally, and blood flow was restored in the left common carotid and internal carotid arteries. The skin wound was closed with a 6‐0 suture. Mice in the sham control group underwent a similar surgery without wire injury. The injured arteries were collected on days 7, 14, and 28 post‐surgery for further studies. Re‐endothelialization of the injured carotid artery was assessed by injection of 5% Evans blue dye (Sigma–Aldrich) through angular vein on day 5 after wire injury. After 10 min of circulation, mice were euthanized and perfused with 4% paraformaldehyde via the left ventricle and then the carotid arteries were collected.

To verify if the phosphorylation of p120 at T310 mediated the function of TTK in vivo, recombinant adeno‐associated virus serotype 9 (AAV‐9) vectors encoding double‐floxed Cre‐inducible empty vector (AAV‐CMV‐DIO‐empty), *TTK* gene (AAV‐CMV‐DIO‐TTK), p120‐T310A mutation gene (AAV‐CMV‐DIO‐p120‐T310A), or p120‐wt gene (AAV‐CMV‐DIO‐p120) were constructed. For the wire injury model, *Myh11‐CreER^T2^* mice were intraperitoneally injected with tamoxifen for five consecutive days to induce Cre expression. On day 7 post‐final injection, the mice were intravenously injected with AAV‐CMV‐DIO‐empty plus AAV‐CMV‐DIO‐p120‐wt, AAV‐CMV‐DIO‐TTK plus AAV‐CMV‐DIO‐p120‐wt, AAV‐CMV‐DIO‐empty plus AAV‐CMV‐DIO‐p120‐T310A mutant, or AAV‐CMV‐DIO‐TTK plus AAV‐CMV‐DIO‐p120‐T310A mutant. Carotid artery wire injury was induced on day 10 post‐virus injection as described above. The mice were euthanized, and the carotid arteries were harvested on day 14 post‐surgery for subsequent experiments.

For in vivo treatment of TTK inhibitor in wire injury model, CFI‐402257 (1, 2, or 4 mg kg^−1^ body weight) (GC18491, GlpBio Technology) or vehicle (90% PEG (06855, Sigma) in ultrapure H2O) was administered orally by means of jelly administration to mice daily for 28 days after carotid wire injury surgery. Carotid artery specimens above were subjected to qRT‐PCR, western blot analysis or fixed and sectioned for histological analysis. To assess potential organ damage in mice treated with inhibitors, different organs (brain, heart, liver, kidney, spleen, and lung) were collected and fixed at the end of the experiment. The fixed tissues were sectioned and stained with H&E.

### Mouse Atherosclerosis Induction and Analysis

To induce atherosclerosis, male *Myh11‐CreER^T2^*/*Rosa26^tdTomato^*/*ApoE^−/−^* mice were euthanized at weeks 0, 4, 8, and 16 post‐feeding on an HFD diet containing 20% milk fat and 0.15% cholesterol (D12079B, Research Diets) as previously described.^[^ ^43^ ^]^ Mice were perfused with PBS through the left ventricle to flush residual blood from the vessels. The aortic arteries were collected for qRT‐PCR and western blotting analyses. The cardiac specimens were fixed and subjected to optimal cutting temperature compound embedding. The frozen aortic roots were serially sectioned for subsequent immunofluorescence staining. *Ttk^ΔSMC^*/*ApoE^−/−^* and their male littermates (*Myh11‐CreER^T2^*/*ApoE^−/−^*; control) were fed on an HFD for 16 weeks. Mice were euthanized and perfused with PBS to flush residual blood from the vessels, and the aortic and cardiac tissue samples were collected. To quantify atherosclerotic lesions in the whole aortic tree, the entire aorta was isolated from the aortic root to the iliac bifurcation with the removal of the perivascular tissues. Subsequently, the aorta was exposed longitudinally, subjected to en face Oil Red O (O9755, Sigma–Aldrich) staining for 15 min at room temperature, and imaged with a digital camera (Nikon, Japan). The frozen aortic root sections were obtained as described above and subjected to HE and Oil Red O staining to assess the size of the lesions in the aortic roots. The lesion area was measured using Image‐Pro Plus software.

To verify if the phosphorylation of p120 at T310 mediated the function of TTK in atherosclerosis, *Myh11‐CreER^T2^*/*ApoE^−/−^* mice were intraperitoneally injected with tamoxifen for five consecutive days to induce Cre expression. On day 7 post‐final injection, the mice were intravenously injected with AAV‐CMV‐DIO‐empty plus AAV‐CMV‐DIO‐p120‐wt, AAV‐CMV‐DIO‐TTK plus AAV‐CMV‐DIO‐p120‐wt, AAV‐CMV‐DIO‐empty plus AAV‐CMV‐DIO‐p120‐T310A mutant, or AAV‐CMV‐DIO‐TTK plus AAV‐CMV‐DIO‐p120‐T310A mutant. HFD diet was fed on day 10 post‐virus injection as described above. The mice were euthanized at week 12 post‐HFD feeding for subsequent experiments.

For in vivo treatment of TTK inhibitor in atherosclerosis, *ApoE^−/−^* mice were fed a HFD for 8 weeks to induce atherosclerotic plaques. These mice were then randomly assigned to different doses of inhibitor CFI‐402257 (1, 2, or 4 mg kg^−1^ body weight) or vehicle (90% PEG in ultrapure H2O) groups with HFD for another 4 weeks. Jellies containing inhibitors or solution controls were administered daily by oral administration.

### Blood Collection and Measurements

After 12–14 h of fasting, peripheral blood samples were collected from mice and serum was collected after centrifugation at 3000 rpm for 15 min. Following the manufacturer's instructions, the levels of total cholesterol, triglycerides, high‐density lipoprotein cholesterol (HDL‐c), and low‐density lipoprotein cholesterol (LDL‐c) were measured with the following assay kits: total cholesterol (A111‐1‐1, Nanjing Jiancheng Bioengineering Institute), triglyceride (A110‐1‐1, Nanjing Jiancheng Bioengineering Institute) HDL‐c (A112‐1‐1, Nanjing Jiancheng Bioengineering Institute), and LDL‐c (A113‐1‐1, Nanjing Jiancheng Bioengineering Institute). Serum blood urea nitrogen (BUN), creatinine (Cr), aspartate aminotransferase (AST), and alanine aminotransferase (ALT) levels were measured using FUJI DRI‐CHEM NX500iVC dry biochemical analyzer.

### Histology and Immunohistochemistry

HE or Oil Red O staining were performed on serial sections (6 µm) of mouse tissue specimens. For immunohistochemistry staining, sections were rehydrated, blocked of endogenous peroxidase, and subjected to antigen retrieval. After blocking with 10% donkey serum, sections were incubated with primary antibody anti‐Ki67 (ab16667, Abcam) at 4 °C overnight and then with biotinylated secondary antibody before treated with EnVision Detection Systems Peroxidase/DAB Rabbit (DAKO). Hematoxylin was used for counter staining. The slides were visualized using an optical microscope (Olympus, Japan).

### Immunofluorescence

For immunofluorescence staining, deparaffinized sections or cryo‐sections were subjected to antigen retrieval by heating the sections in PH 6.0 citrate buffer at 95 °C for 20 min, followed by permeabilization with 0.5% Triton X‐100 in PBS for 10 min and blocking with 10% donkey serum for 30 min at room temperature. Sections were then incubated with primary antibodies overnight at 4 °C, followed by Alexa Fluor 488 or 594 conjugated secondary antibodies (1:100; Invitrogen) incubation at room temperature for 1 h. The following primary antibodies were used: anti‐TTK (1:50, A2500, Abconal), anti‐α‐SMA (1:100, ab21027, Abcam), anti‐CD31 (1:50, AF3628, R&D Systems), ChromoTek RFP Monoclonal antibody (5F8) (1:1000, Proteintech). The nuclei were visualized by staining with DAPI (C1005, Beyotime) for 10 min. Fluorescent images were captured using a confocal laser scanning microscopy (Nikon, Tokyo, Japan). The percentage of TTK**^+^** tdTomato**^+^** / tdTomato**^+^** (double positive cell number divided by total tdTomato positive cell number) in neointima or atherosclerotic plaques was used to display the expression change of TTK.

### Cell Culture

Primary mouse VSMCs were isolated from 8‐week‐old male C57BL/6 mice by DNase, elastase, and collagenase digestion as described previously.^[^ ^40^ ^]^ The purity of VSMCs was confirmed by immunofluorescence staining for α‐SMA. Isolated VSMCs were cultured in growth media SmGM‐2 (Lonza) containing 5% fetal bovine serum (FBS). Cells at passages four to eight were used for further experiments. Mouse aortic vascular smooth muscle cell line (MOVAS, ATCC, CRL‐2797) was cultured in 10% FBS. The MOVAS cells were used in Figures 2E,F,J, and S3D (Supporting Information). All cells were cultured in a humidified 5% CO2 incubator at 37 °C.

### RNA Isolation and Quantitative Real‐Time PCR

Total RNA was extracted from VSMCs, arteries, or plaques with RNA isoplus kit (Takara, Japan). cDNAs were synthesized with the cDNA Synthesis Kit (Takara). Real‐time PCR was performed using SYBR Premix Ex TaqTM Kit (RR420A, Takara) according to the manufacturer's instructions. The mRNA expression levels were normalized to β‐actin. The primers used are listed in Table S3 (Supporting Information).

### Western Blot Analysis

Cell and tissue samples were lysed in RIPA buffer (PM0013B, Beyotime) containing freshly added protease cocktail inhibitors. Equal amounts of total protein in each group were resolved and separated on SDS‐PAGE, and subsequently transferred onto PVDF membranes (EMD Millipore, Billerica, MA, USA). After blocking with 5% skim milk or 5% BSA in TBST, the membranes were incubated at 4 °C overnight with primary antibodies against TTK (AF6028, R&D Systems), TTK (A2500, Abclonal), IRF1 (#8478, CST), E2F4 (10923‐1‐AP, Proteintech), C/EBP β (sc‐7962, Santa Cruz), α‐SMA (ab5694, Abcam), SM22α (ab10135, Abcam), calponin (ab46794, Abcam), p120‐catenin (12180‐1‐AP, Proteintech), p‐p120 (Thr310) (bs‐12990R, Bioss), HA (ab9110, Abcam), β‐catenin (51067‐2‐AP, Proteintech), N‐cadherin (ab18203, Abcam), SRF (16821‐1‐AP, Proteintech), MYOCD (SAB4200539, Sigma‐Aldrich), Cre Recombinase (#15036, CST), H3 (AF0009, Beyotime), β‐tubulin (AC015, Abclonal) and β‐actin (AC026, Abclonal). After incubation with horseradish peroxidase (HRP)‐conjugated secondary antibodies of the appropriate species for 1 h at room temperature, protein bands on the membrane were visualized on a BioSpectrum imaging system (UVP, USA) using ECL solution, and the expression levels of proteins were evaluated with ImageJ software.

### siRNA Transfection

Small interfering RNAs (siRNAs) against TTK, CEP55, NCAPH, IRF1, E2F4, C/EBPβ, and a scrambled siRNA were designed and synthesized by RiboBio (Guangzhou, China). The siRNAs were transfected into cells in vitro following the manufacturer's protocol of Lipofectamine 3000 (Invitrogen). The silencing efficiency was verified by PCR and Western blot analysis at 24 h after transfection. The sequence of siRNAs is listed in Table S4 (Supporting Information).

### DNA Constructs and Luciferase Reporter Assays

An overexpression vector containing full‐length cDNA of IRF1 was prepared by PCR and cloned into the control vector. A sample of the TTK promoter of ≈2000 bp was PCR amplified and inserted into the pGL3‐basic vector (Promega). Mutant vectors of TTK promoter were cloned into luciferase reporter with mutation of the IRF1 binding sites 1 or 2, respectively. Co‐transfection of the luciferase reporter constructs with an IRF1 overexpression vector or empty vector was conducted. The cell lysates were used for luciferase activity assays with a microplate reader in accordance with the manufacturer's instructions.

### Chromatin Immunoprecipitation (ChIP)

ChIP assays were performed following the previously reported protocols with minor modifications.^[^ ^40^ ^]^ VSMCs were treated with 20 ng mL^−1^ PDGF‐BB or 50 µg mL^−1^ ox‐LDL for 24 h and fixed in 1% formaldehyde for 10 min to cross‐link chromatin. Cells were quenched with 125 mm glycine for 5 min at room temperature, followed by washing with ice‐cold PBS. The lysates were prepared following the manufacturer's instructions and sonically sheared into DNA fragments. Immunocomplexes were precipitated by incubating the samples with anti‐IRF1 antibodies (CST) or IgG overnight. The immunoprecipitated DNA was extracted using a DNA purification kit. The target regions of TTK promoter were amplified using PCR with the following primers: 5′‐TGACAGGTCGTCTGTTGTCG‐3′ (forward) and 5′‐AGCAGTGGCAGACTGTGAAA‐3′ (reverse).

### Co‐Immunoprecipitation

Cells or tissue lysates were prepared using NP40 lysis buffer (Beyotime, China) containing phenylmethylsulfonyl fluoride and a phosphatase inhibitor cocktail. The samples were centrifuged, and the supernatant was incubated with anti‐HA (ab9110, Abcam), anti‐p120 (12180‐1‐AP, Proteintech), anti‐Irf2bpl (sc‐514772, Santa Cruz) anti‐N‐cadherin (ab18203, Abcam), anti‐β‐catenin (51067‐2‐AP, Proteintech), or anti‐SRF (16821‐1‐AP, Proteintech) antibodies overnight at 4 °C. Next, the samples were incubated with protein A&G agarose beads (Beyotime, China) for 2 h to precipitate immune complexes. The samples were subjected to washing and elution, following the manufacturer's protocols. Immunoblotting was performed with the indicated antibodies.

### Immunoprecipitation and Tandem Mass Spectrometry Analysis

The VSMCs transfected with HA‐tagged TTK constructs were lysed. Proteins were harvested for immunoprecipitation using the anti‐HA antibodies and subjected to sodium dodecyl sulfate‐polyacrylamide gel electrophoresis. The gel was stained with Coomassie blue. The lysates were digested with trypsin and subjected to liquid chromatography‐tandem mass spectrometry (LC‐MS/MS) analysis. LC‐MS/MS analysis was performed using an Orbitrap Fusion mass spectrometer (Thermo Fisher Scientific) coupled with an EASY‐nLC 1000 UPLC system (Thermo Fisher Scientific), following the manufacturer's instructions.

### Phosphoproteomic Analysis

Phosphoproteomic analysis was performed following the previously described methods.^[^ ^44^ ^]^ Briefly, cells were lysed in lysis buffer (8 m urea, 1% protease inhibitor, and 1% phosphatase inhibitor) using ultrasonication. The lysates were centrifuged to remove debris. The protein concentrations in the supernatant were quantified using the bicinchoninic acid protein assay kit (Beyotime, China). After digestion with trypsin overnight (trypsin‐to‐protein mass ratio of 1:50), the proteins were reduced with 5 mm dithiothreitol for 30 min at 56 °C and alkylated with 11 mm iodoacetamide for 15 min at room temperature in the dark. The peptide samples were desalted using Strata X C18 (Phenomenex), vacuum dried, and labeled using a tandem mass tag kit, following the manufacturer's instructions. Next, the peptides were fractionated using high pH reverse‐phase high‐performance liquid chromatography with an Agilent 300 Extend C18 column (5 µm particles, 4.6 mm ID, 250 mm length). The peptides were reconstituted in the enrichment buffer (50% acetonitrile/6% trifluoroacetic acid). Iron‐immobilized metal ion affinity chromatography microspheres were used to enrich the phosphopeptides for subsequent LC‐MS/MS analysis. The peptides were separated using an EASY‐nLC 1200 UPLC system, ionized using a nanospray ion source, and analyzed using an Orbitrap Exploris 480 mass spectrometer. The raw data was processed using MaxQuant (v.1.6.2.6) integrated with the Andromeda search engine. Tandem mass spectra were searched against the Mus_musculus_10090_SP_20210721.fasta database concatenated with the reverse decoy database. The mass errors for precursor ions and fragment ions were set as 10 ppm and 0.02 Da, respectively. Carbamidomethylation (C) was specified as a fixed modification, while acetylation (protein N‐terminus), oxidation (M), and phosphorylation (STY) were specified as variable modifications. The false discovery rates (FDRs) for proteins, peptides, and peptide spectrum match level was set to < 1%.

### Identification of DEGs in GEO Datasets

The raw data of the mRNA expression datasets GSE70410, GSE40637, GSE164050, and GSE69637 were downloaded from the GEO database in the National Center for Biotechnology Information (http://www.ncbi.nlm.nih.gov/gds/) and analyzed with the R software package. Unqualified data were transformed and filtered. The data were subjected to calibration, standardization, and transformation. Based on the type of data, the DEGs were identified using the “Limma” package of R software. Adjusted *p*‐values were calculated using the FDR. The DEGs of each dataset are shown in Data S1 (Supporting Information).

### Wound Healing Assays

VSMCs were plated in DMEM with 10% FBS into 6‐well plates. When at a suitable confluence, the cells were treated with DMEM containing 5 µg mL^−1^ mitomycin‐C overnight. Then, linear wounds through the center of the wells were scratched using a 200‐µL pipette tip. After washing the floating cells, different stimulations were given in DMEM for 24 h. The images of the wounds were captured at specified time using a microscope with a digital camera. The migration rates were analyzed using ImageJ.

### Transwell Assays

After serum starvation in DMEM overnight, VSMCs were digested with trypsin and resuspended in DMEM. The cell suspension (≈5.0 × 10^4^ cells in 200 µL DMEM per well) was added to the upper chamber of the transwell chamber containing polycarbonate filters with an 8 µm pore size. Different stimuli dissolved in 500 µL DMEM were added to its lower chamber. After incubated at 37 °C for 6 h in 5% CO2 incubator, the filters were fixed at 4% PFA and staining with 10% crystal violet for 10 min. Then, the cells remaining on the upper surfaces of the filters were gently rubbed off using a cotton swab. The cells that migrated to the undersides of the filters were photographed using a microscope with a digital camera and counted randomly.

### Cell Proliferation Assays

VSMCs were seeded in DMEM with 10% FBS into 24‐well plates. After starved in DMEM overnight when their density was appropriate, the cells were treated with different stimulations in DMEM for 24 h. The cells were fixed with 4% PFA for 30 min, permeabilized with 0.5% Triton X‐100 for 10 min and blocked with 5% donkey serum for 30 min. Then, the cells were incubated with Ki67 antibody (1:100, GB111141, Servicebio) overnight at 4 °C. The secondary antibody conjugated with fluorescein was used to label primary antibody for 1 h and images of the cells were captured using a fluorescence microscope.

### Collagen Gel Contraction Assay

The collagen gel contraction assay was performed by using Cell Contraction Assay Kit (Cell Biolabs). The contractile ability of VSMCs was assessed following the manufacturer's protocol. Briefly, VSMCs were harvested and resuspended in culture medium at a density of 5 × 10^5^ cells mL^−1^. A collagen lattice was prepared by mixing the cell suspension and ice‐cold collagen gel solution in a volume ratio of 1:4. After that, 0.5 mL of the cell‐collagen mixture was added to a 24‐well plate followed by incubated for 1 h at 37 °C. Then, 1.0 mL of culture medium was added on top of each collagen gel lattice after collagen polymerization. The images of the plates were captured 24 h later. The NIH ImageJ software was used to analyzed the area of the gel in each well.

### Blood Pressure Measurements

A CODA Mouse & Rat Tail‐Cuff Blood Pressure System (Kent Scientific Co., Connecticut, USA) was used to detect the blood pressure levels.^[^ ^8^ ^]^ Briefly, mice were placed in tail‐cuff restrainers situated on a warmed surface and should be habituated to the device before the measurement of blood pressure. A quiet, dark environment was maintained to ensure reliable measurements within the parameters of this technology. One set of six measurements were obtained for each mouse, and mean blood pressure was calculated.

### Statistical Analysis

All statistical analyses were performed using GraphPad Prism 9.0 software. Data are expressed as mean ± SEM. The sample size (*n*) for each statistical analysis is provided in the figure legends. The normal distribution of data was examined using the Shapiro–Wilk test. Means between two groups were compared using an unpaired two‐tailed Student's *t*‐test (for normally distributed data with similar variances) or the unpaired *t*‐test with Welch's correction (for normally distributed data with significantly different variances). Non‐normally distributed data between two groups were compared using the Mann–Whitney test. Means between more than two groups were assessed for similar variance using the Brown–Forsythe test and compared using one‐way ANOVA (for data with similar variance) or Welch's ANOVA (for data with dissimilar variance). A general linear model was used to study the correlation between two variables. Two‐tailed *p* values < 0.05 were considered statistically significant.

## Conflict of Interest

The authors declare no conflict of interest.

## Supporting information



Supporting Information

Supporting Information

Supporting Information

Supporting Information

## Data Availability

The data that support the findings of this study are available on request from the corresponding author. The data are not publicly available due to privacy or ethical restrictions.
