# Supplementary material for: TTK Inhibition Alleviates Postinjury Neointimal Formation and Atherosclerosis
Source: Adv Sci (Weinh). 2024 Dec 24;12(6):2409250. doi: 10.1002/advs.202409250 (PMC11809377; doi:10.1002/advs.202409250)
Supplement: Supplementary file 1 — Supporting Information [file ADVS-12-2409250-s003.docx]

**Supporting Information**

**TTK Inhibition Alleviates Postinjury Neointimal Formation and Atherosclerosis**

*Jie-Hong Wu, Yu-Xiao Liu, Jia-Bin Zong, Min Qiu, Yi-Fan Zhou, Ya-Nan Li, Tuersun Aili, Xin-Ran Zhao, and Bo Hu**


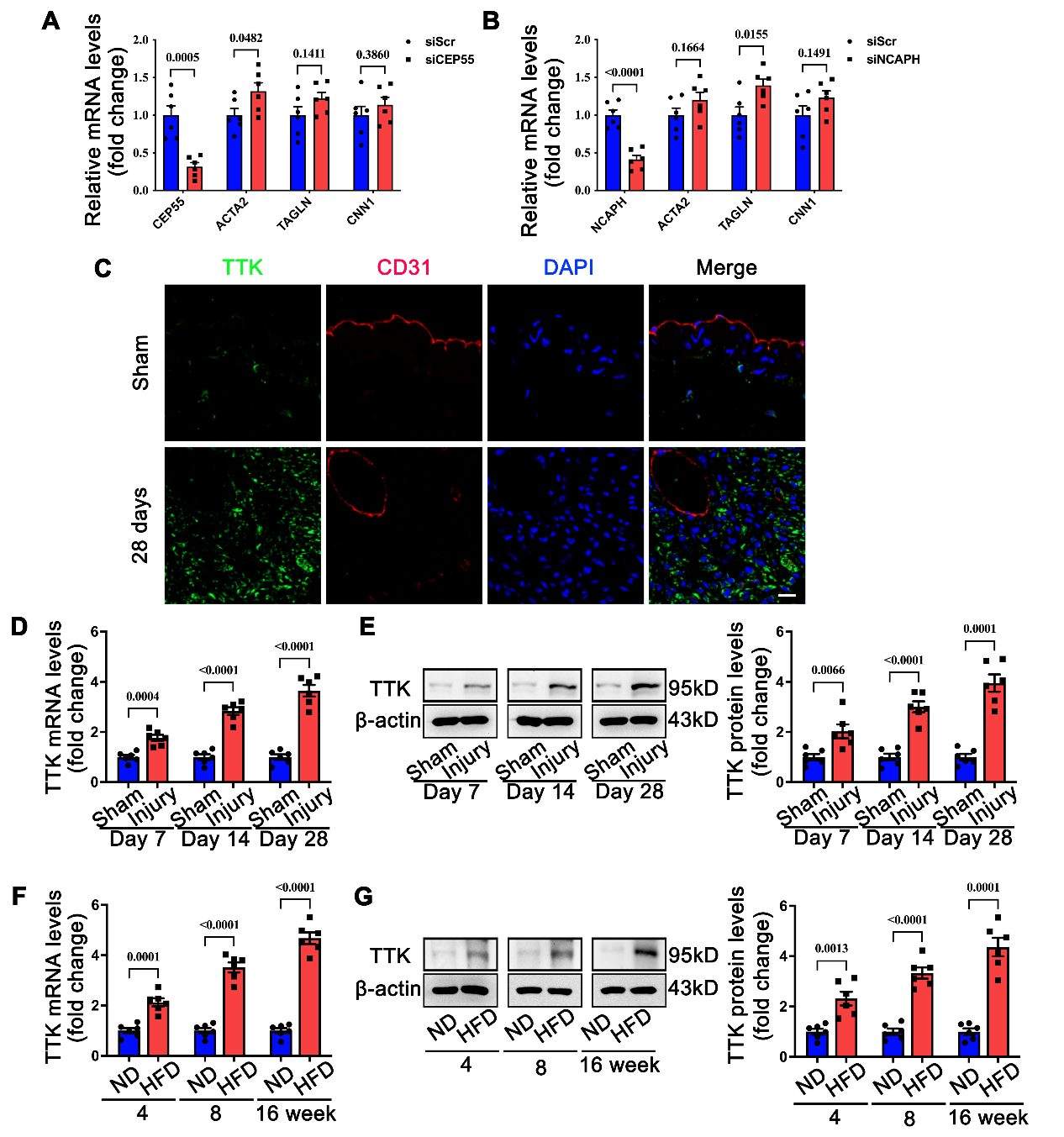


**Figure S1. TTK is overexpressed during neointima formation in vascular injury and atherosclerosis.**

A) qRT-PCR analysis of the relative mRNA level of CEP55, ACTA2, TAGLN, and CNN1 in VSMCs transfected with scrambled siRNA or CEP55-specific siRNA (*n* = 6). B) qRT-PCR analysis of the relative mRNA level of NCAPH, ACTA2, TAGLN, and CNN1 in VSMCs transfected with scrambled siRNA or NCAPH -specific siRNA (*n* = 6). C) Representative immunofluorescence staining of TTK (green) and CD31 (red) in the sham-operated or wire-injured carotid sections of *Myh11-CreER^T2^*/*Rosa26^tdTomato^* mice on day 28 post-surgery. Scale bar = 10 μm. D,E) Relative levels of TTK mRNA D) and protein E) in mouse carotid arteries on days 7, 14, and 28 post-sham or wire injury operation. Protein levels were normalised to β-actin levels (*n* = 6). F,G) Relative levels of TTK mRNA F) and protein G) in the aortic arteries of *ApoE^−^*^/^*^−^* mice fed on HFD for 0, 4, 8, and 16 weeks. Protein levels were normalised to β-actin levels (*n* = 6). Data are presented as the mean ± SEM; unpaired *t*‐test.


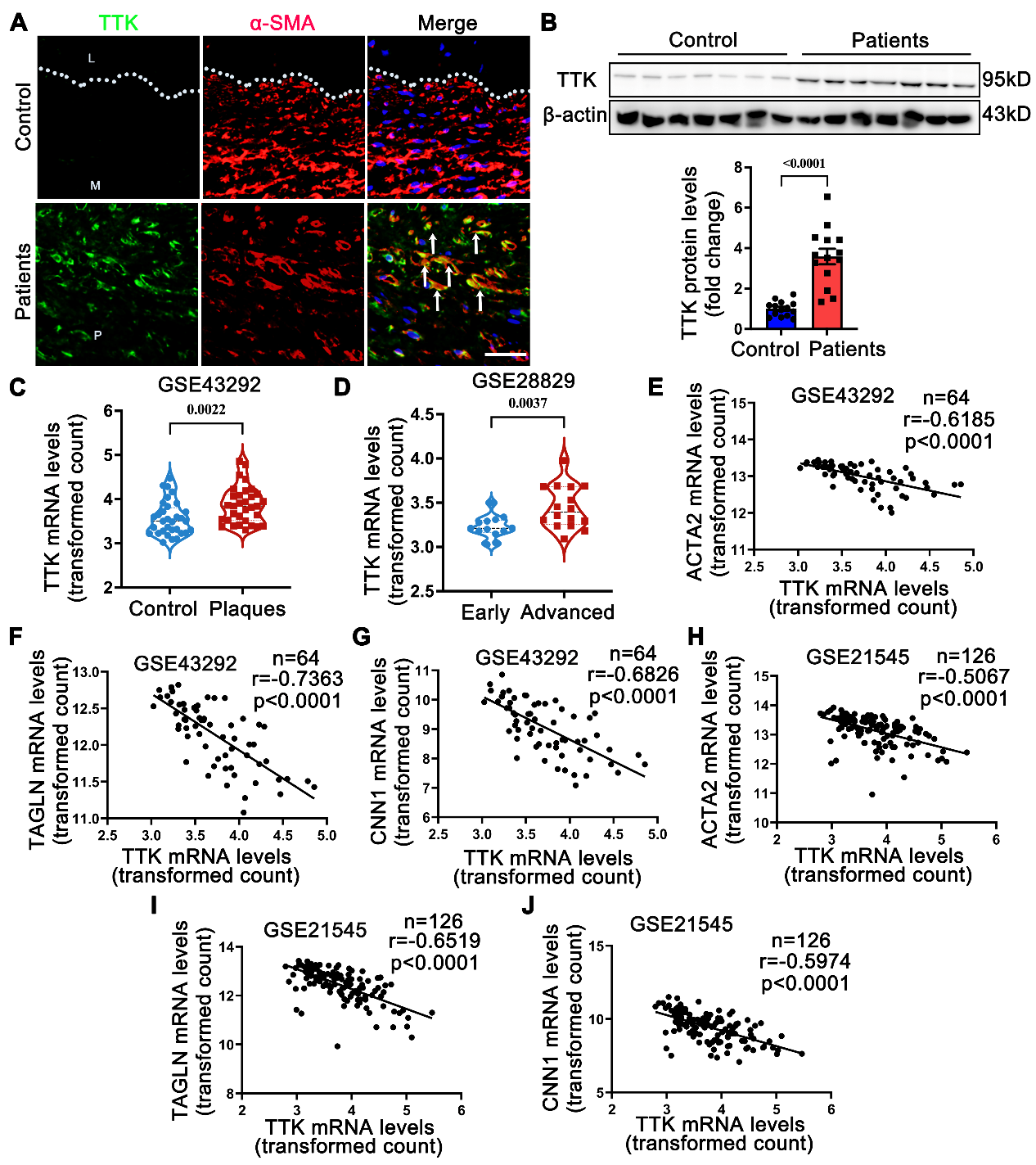


**Figure S2. The expression pattern between TTK and SMC contractile markers in human atherosclerotic plaques.**

A) Representative immunofluorescence images of TTK (green) and α-SMA (red) in the healthy human artery and carotid atheroma sections. Nuclei were stained with 4′,6-diamidino-2-phenylindole (DAPI) (blue). The dotted lines indicate the border between the lumen (L) and the media (M). White arrows indicate TTK^+^ α-SMA^+^ cells. Scale bar = 50 μm. P, plaque. B) Representative western blotting analysis and quantification of TTK protein levels in healthy human arteries and carotid atheroma. Protein levels were normalised to β-actin levels (*n* = 14). C) The TTK mRNA levels in human non-atherosclerotic arteries and atherosclerotic plaques from data curated in the GEO dataset GSE43292. D) The TTK mRNA levels in early and advanced human atherosclerotic plaques from data curated in the GEO dataset GSE28829. E-J) Negative correlations between the mRNA levels of TTK and SMC contractile markers (ACTA2, TAGLN, and CNN1) in two independent human atherosclerotic GEO datasets (GSE43292 and GSE21545). Data are presented as the mean ± SEM; unpaired *t*‐test.

**
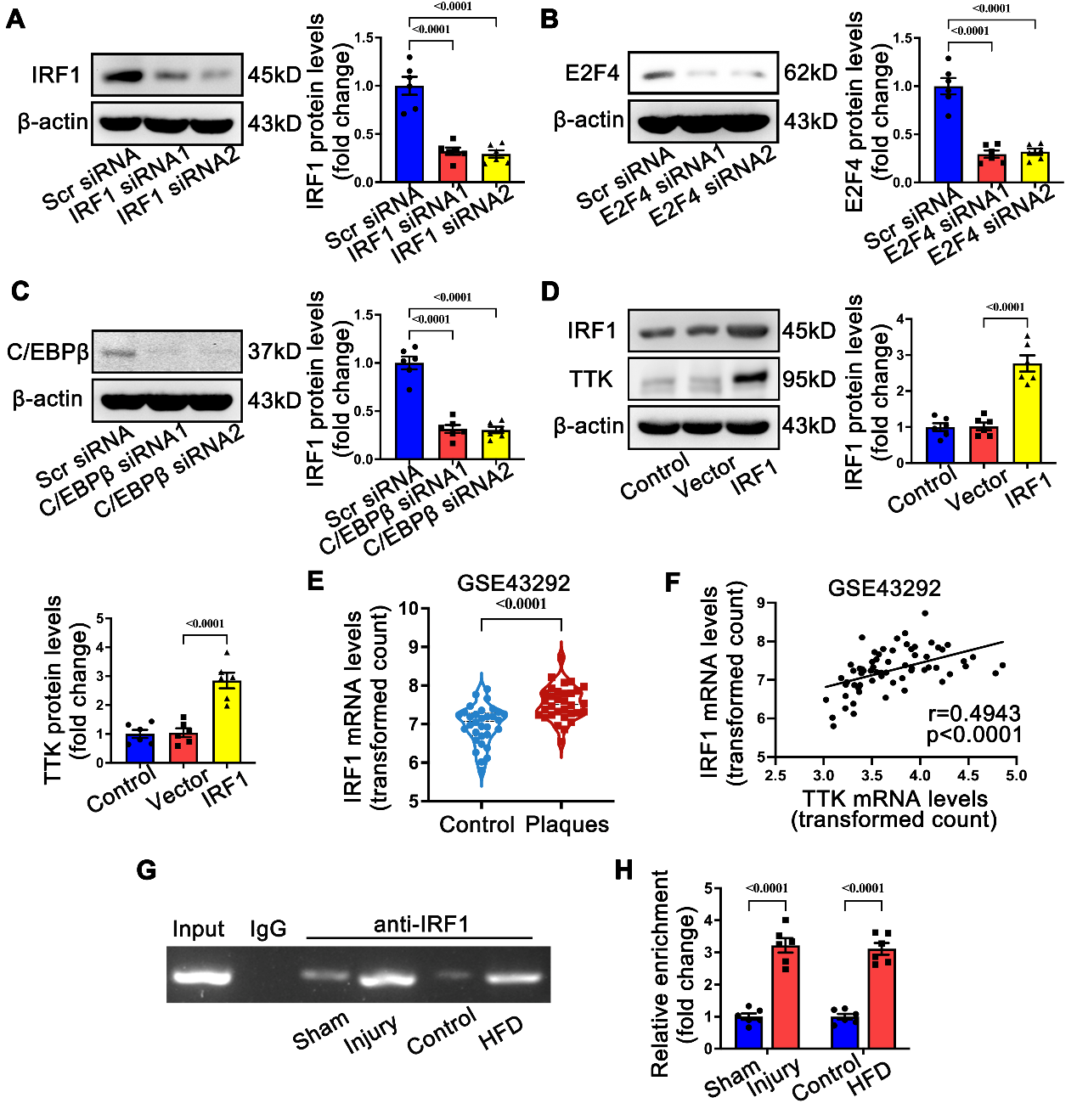
**

Figure S3. IRF1 upregulates TTK transcription in VSMCs upon pathological stimulation.

A–C) Western blotting validation and quantification of the knockdown efficiency of small interfering RNAs (siRNAs) against IRF1 A), E2F4 B), and C/EBPβ C) in VSMCs. Protein levels were normalised to β-actin levels (*n* = 6). D) Representative western blotting and quantification of IRF1 and TTK protein levels in MOVAS cells transfected with empty vector or IRF1-encoding constructs. Protein levels were normalised to β-actin levels (*n* = 6). E,F) The IRF1 mRNA levels in human non-atherosclerotic arteries and atherosclerotic plaques from data curated in the Gene Expression Omnibus dataset GSE43292 are shown in E). The IRF1 mRNA levels were positively correlated with the and TTK mRNA levels in human carotid arteries in GSE43292 in F). G,H) Chromatin immunoprecipitation (ChIP) assay G) and quantification H) demonstrated that carotid injury and atherosclerosis promoted the binding of IRF1 to the TTK promoter (*n* = 6). IgG lane: negative control. Data are presented as the mean ± SEM; unpaired *t*‐test, one‐way ANOVA.

**
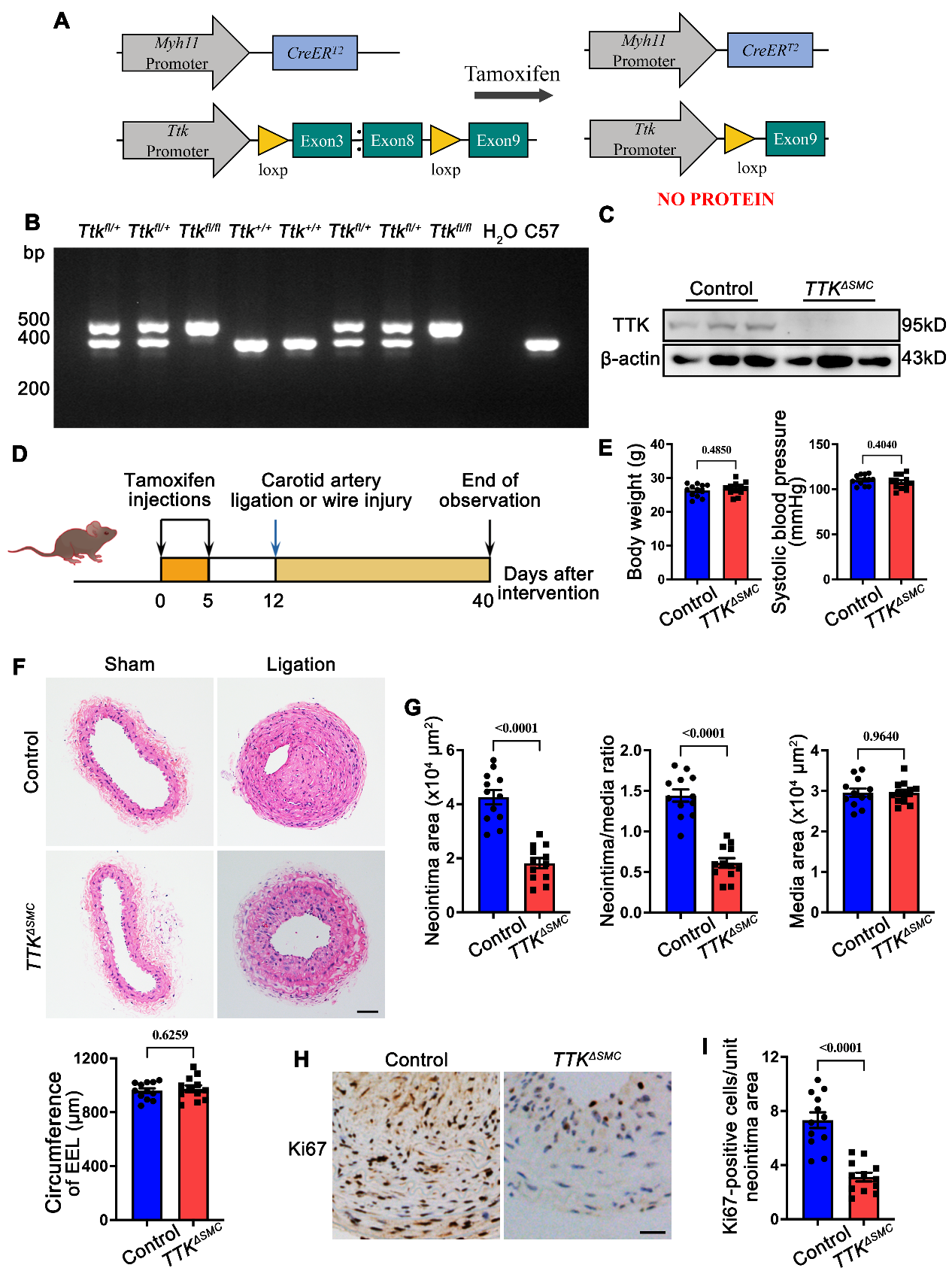
**

**Figure S4. TTK deficiency in VSMC suppresses neointima formation in the carotid artery ligation models.**

A) Schematic view of the generation of *Ttk^ΔSMC^* mice. B) Genotyping of *Ttk^ΔSMC^* mice using polymerase chain reaction (PCR) analysis. C) Representative western blotting of TTK in carotid arteries from control and *Ttk^ΔSMC^* mice. Protein levels were normalised to β-actin levels. D) Schematic illustration of the experimental design for evaluating the effect of TTK deficiency in VSMCs on neointima formation in control and *Ttk^ΔSMC^* mice. E) Body weight and systolic blood pressure of control and *Ttk^ΔSMC^* mice (*n* = 12). F) Representative HE-stained sections of sham-operated and ligated carotid arteries from control and *Ttk^ΔSMC^* mice on day 28 post-surgery. Scale bar = 50 μm. G) Quantitative analysis of the neointima area, neointima-to-media ratio, media area and EEL circumference in the histological sections of ligated carotid arteries (*n* = 12). H,I) Representative Ki67 immunohistochemistry staining H) and corresponding quantification of Ki67-positive cells in the neointima I) in sections of sham-operated and ligated carotid arteries from control and *Ttk^ΔSMC^* mice on day 28 post-surgery (*n* = 12). Scale bar = 20 μm. Data are presented as the mean ± SEM; unpaired *t*‐test.


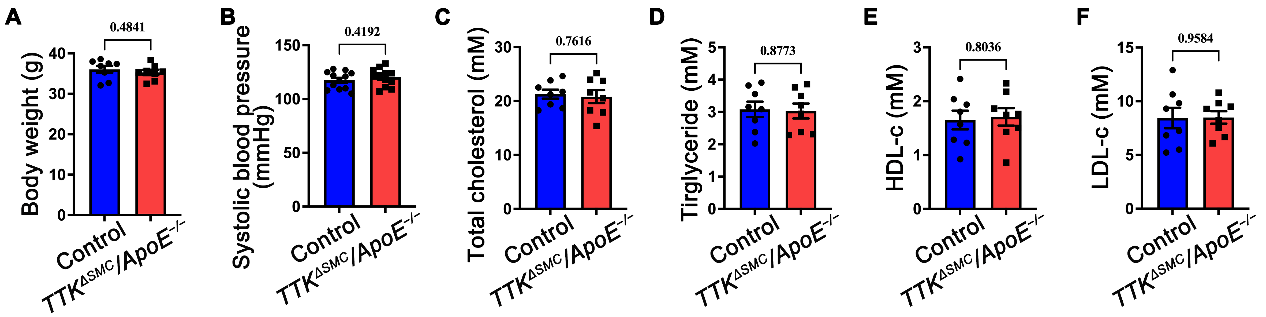


**Figure S5. TTK deficiency does not affect the body weight and lipid profiles of high-fat diet (HFD)-fed *ApoE^−/−^* mice.**

A–F) Body weight, systolic blood pressure, serum total cholesterol, triglyceride, high-density lipoprotein-cholesterol (HDL-c), and low-density lipoprotein-cholesterol (LDL-c) levels in control and *Ttk^ΔSMC^*/*ApoE^−/−^* mice (*n* = 8). Data are presented as the mean ± SEM; unpaired *t*‐test.


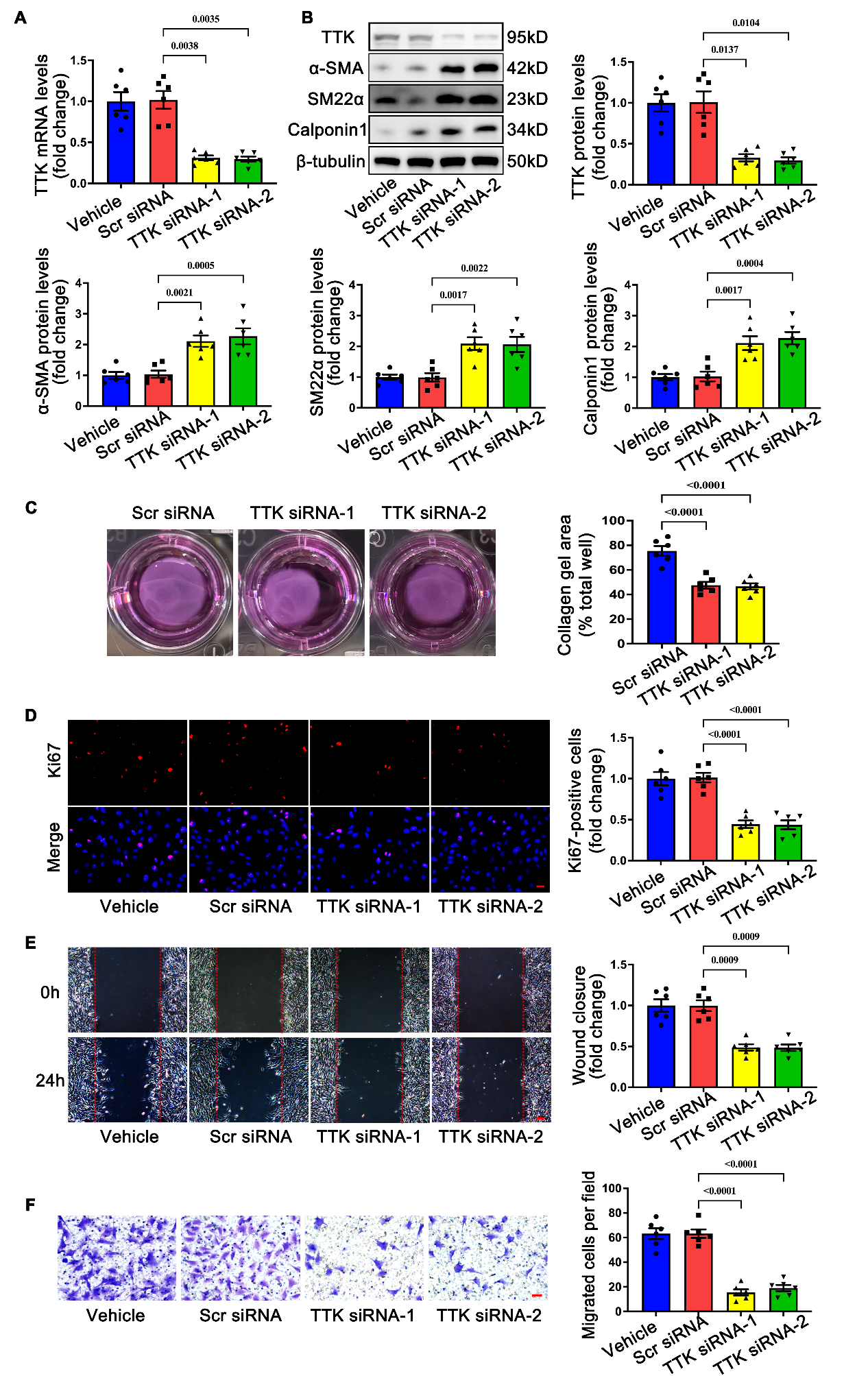


**Figure S6. TTK knockdown inhibits the phenotypic switching of VSMCs in vitro.**

A) VSMCs were transfected with scrambled siRNA (scrRNA) or TTK-specific siRNAs (siRNA-1 or siRNA-2). qRT-PCR analysis of the relative mRNA level of TTK in VSMCs transfected with scrambled siRNA or TTK-specific siRNAs (*n* = 6). B) Representative western blotting and quantification of TTK, α-SMA, SM22α, and calponin1 in VSMCs transfected with scrambled siRNA or TTK-specific siRNAs. Protein levels were normalised to β-tubulin levels (*n* = 6). C) Representative images and quantification of collagen gel contraction containing VSMCs transfected with scrambled siRNA or TTK-specific siRNAs (*n* = 6). D) Representative Ki67 immunofluorescence images and corresponding quantification of Ki67-positive cells in VSMCs transfected with scramble siRNA or TTK-specific siRNAs (*n* = 6). Scale bar = 20 μm. E,F) Representative images of VSMC migration analysed using the wound healing assay E) and transwell assay F) and the quantification of migration areas and migrated cells in VSMCs transfected with scramble siRNA or TTK-specific siRNAs (*n* = 6). Scale bar = 100 μm (upper) or 50 μm (lower). Data are presented as the mean ± SEM; one‐way ANOVA.


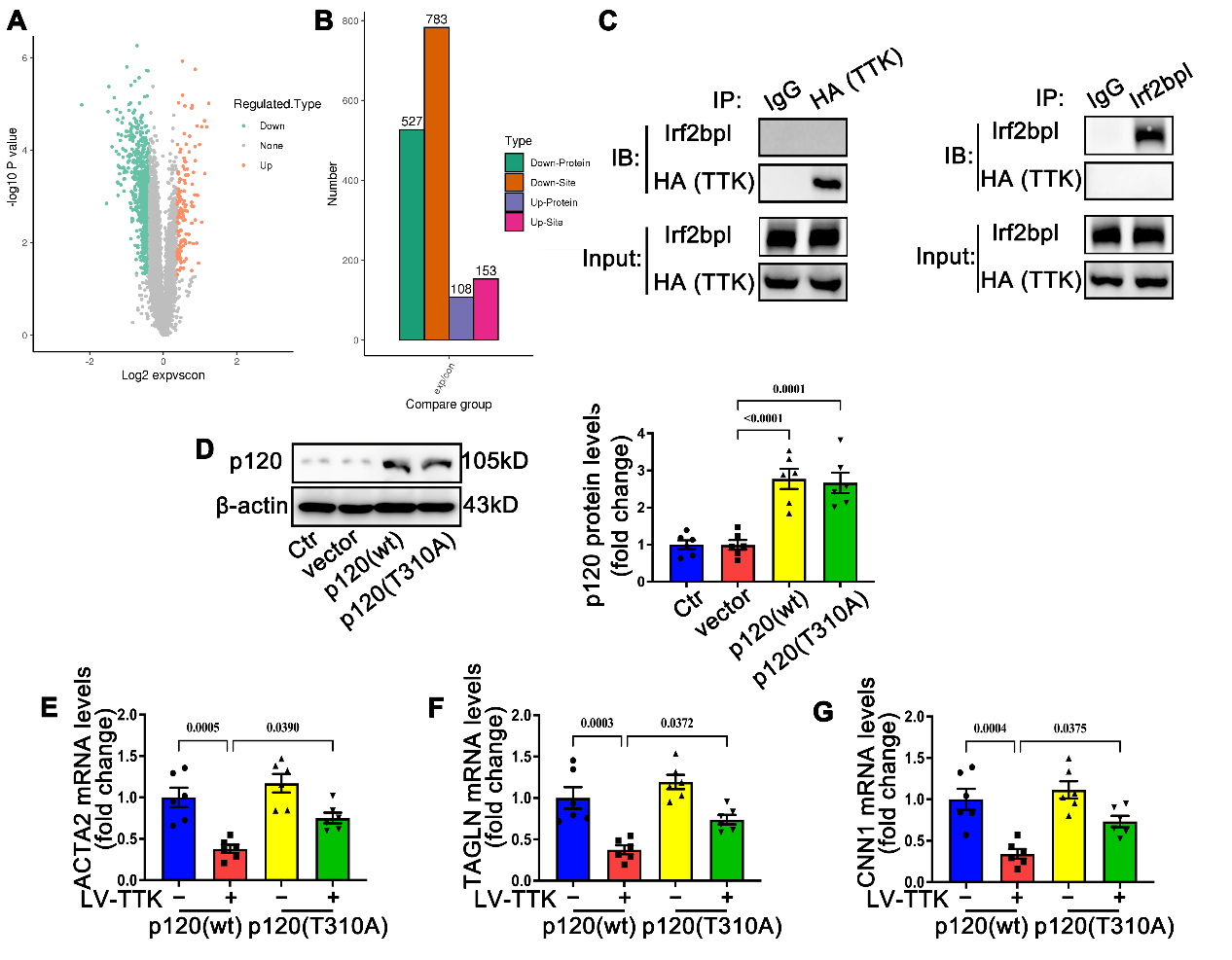


**Figure S7. Phosphoproteomic analysis of the downstream molecular mechanisms underlying the regulatory effects of TTK on the phenotypic switching of VSMCs.**

A) The volcano plot of differentially expressed phosphorylation sites between hemagglutinin (HA)-tagged TTK construct-transfected and control vector-transfected groups determined using phosphoproteomic analysis. B) Differentially expressed phosphorylation sites and corresponding proteins between HA-tagged TTK construct-transfected and control vector-transfected groups. C) Lysates of VSMCs transfected with the HA-tagged TTK lentivirus were immunoprecipitated with anti-HA antibodies, and the precipitates were analysed using immunoblotting with anti-Irf2bpl antibodies (left). Lysates of VSMCs transfected with the HA-tagged TTK lentivirus were immunoprecipitated with anti-Irf2bpl antibodies, and the precipitates were analysed using immunoblotting with anti-HA antibodies (right). D) Representative western blotting and quantification of p120 protein levels in VSMCs transfected with vector, wild-type p120 (p120-wt), or mutant p120 (p120-T310A). Protein levels were normalised to β-actin levels (*n* = 6). E-G) Relative mRNA levels of ACTA2 (E), TAGLN (F), and CNN1 (G) in VSMCs co-transfected with empty vector or LV-TTK and p120-wt or p120-T310A (*n* = 6). Data are presented as the mean ± SEM; one‐way ANOVA.


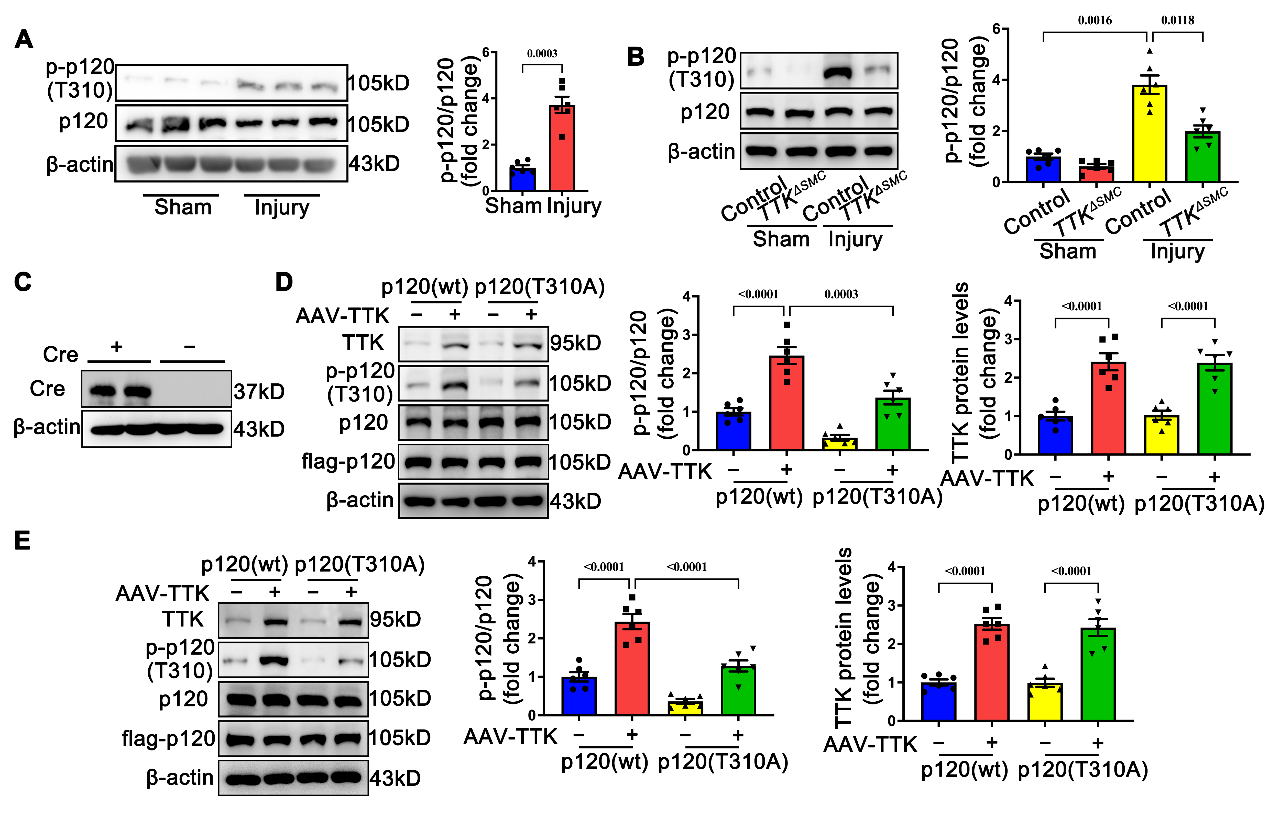


**Figure S8. Validation of the TTK/phosphorylated p120-catenin (T310) pathway in vivo.**

A) Representative western blotting and quantification of p-p120 (T310) in wire-injured carotid arteries of C57 mice (*n* = 6). B) Representative western blotting and quantification of p-p120 (T310) in wire-injured carotid arteries from control and *Ttk^ΔSMC^* mice on day 28 (*n* = 6). C) Representative western blotting of Cre in carotid arteries from mice with or without *Myh11-CreER^T2^*. D) *Myh11-CreER^T2^* mice were intraperitoneally injected with tamoxifen for 5 consecutive days to induce Cre expression. On day 7 post-final injection, the mice were intravenously injected with different virus. Carotid artery wire injury was induced on day 10 post-virus injection. The mice were euthanized, and the carotid arteries were harvested on day 14 post-surgery for the quantification of p-p120 (T310) and TTK protein levels (*n* = 6). E) *Myh11-CreER^T2^*/*ApoE^−/−^* mice were intraperitoneally injected with tamoxifen for 5 consecutive days to induce Cre expression. On day 7 post-final injection, the mice were intravenously injected with different virus. Mice were fed on a high-fat diet (HFD) on day 10 post-virus injection. The mice were euthanized at 12 weeks post-HFD feeding, and the aortic arteries were harvested for the quantification of p-p120 (T310) and TTK protein levels (*n* = 6). Data are presented as the mean ± SEM; unpaired *t*‐test, one‐way ANOVA.


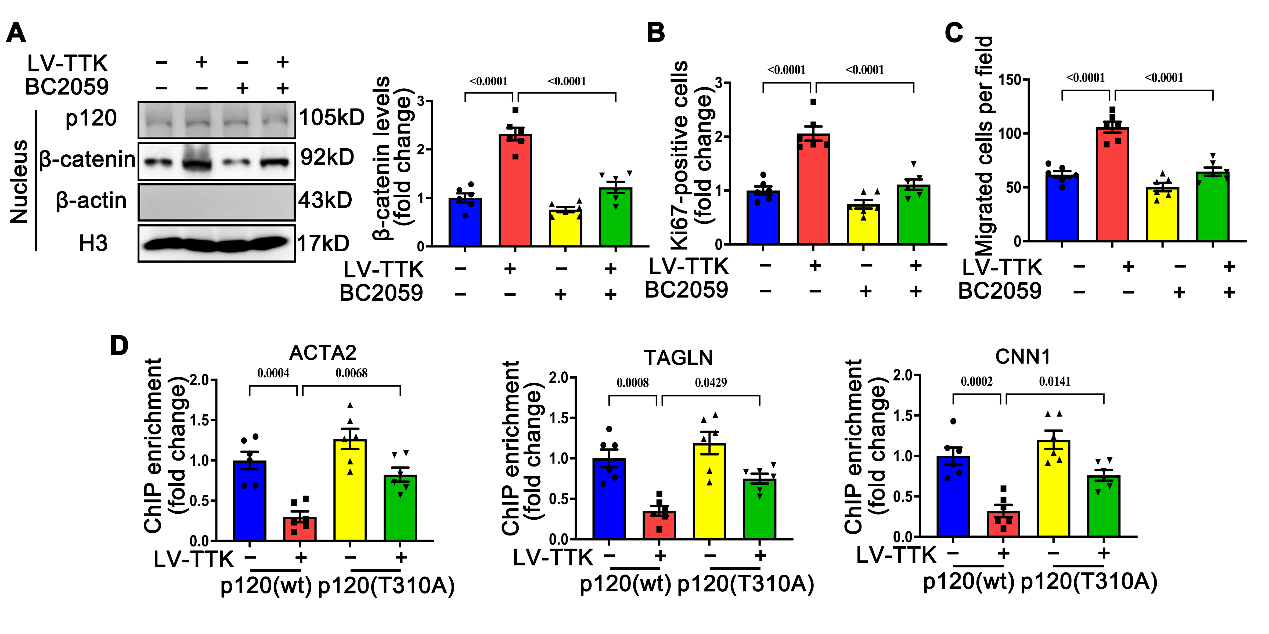


**Figure S9. TTK-induced phenotypic switching of VSMCs is dependent on the nuclear accumulation of β‐catenin in vitro.**

A) Representative western blotting and quantification of nuclear p120 and β-catenin protein levels in VSMCs transfected with vector or LV-TTK in the presence or absence of 40 nM BC2059. Protein levels were normalised to H3 levels (*n* = 6). B,C) Quantification of Ki67 immunofluorescence staining B) and transwell assay results C) of VSMCs transfected with vector or LV-TTK in the presence or absence of 40 nM BC2059 (*n* = 6). D) ChIP analysing the binding of SRF to promoters of the ACTA2, TAGLN and CNN1 promoter in VSMCs co-transfected with empty vector or LV-TTK and p120-wt or p120-T310A (*n* = 6). Data are presented as the mean ± SEM; one‐way ANOVA.


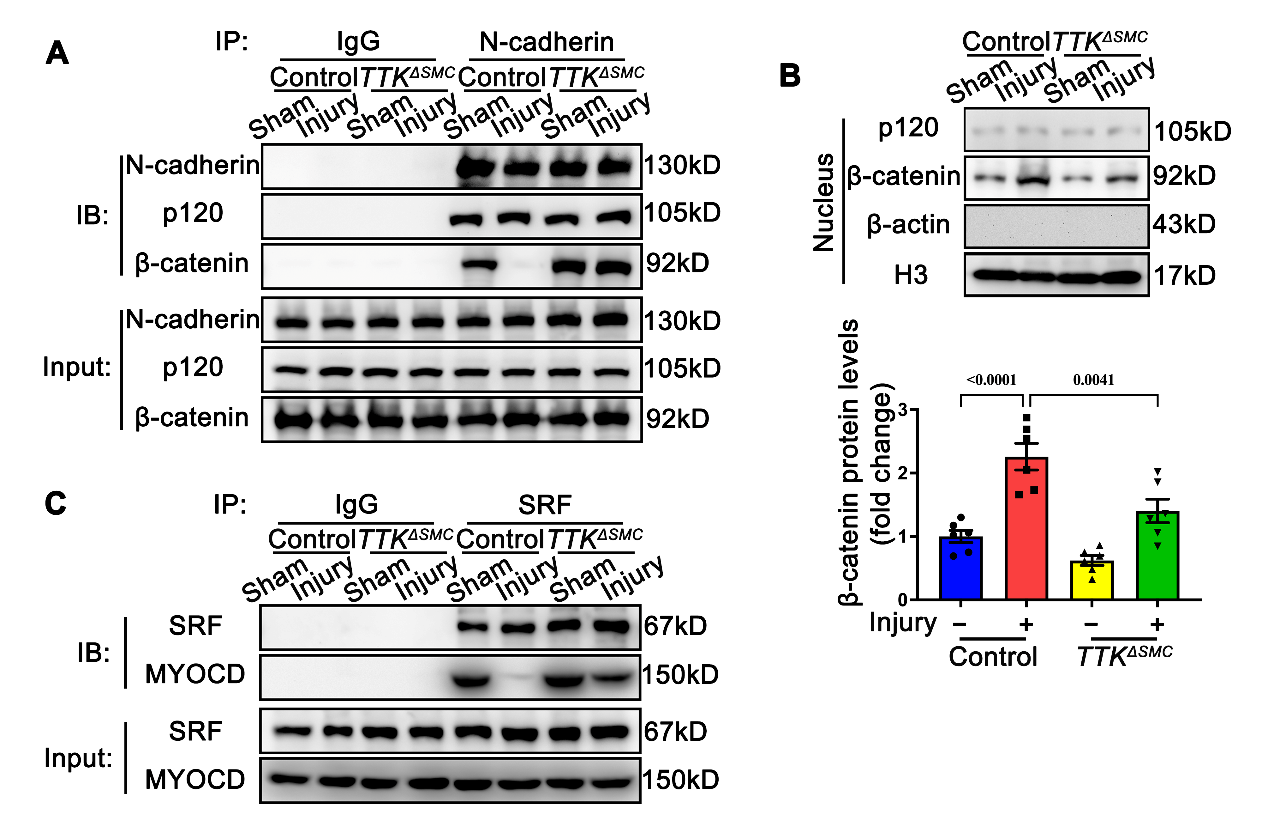


**Figure S10. Verification of the downstream mechanisms of the TTK-p120 (T310) pathway using SMC-specific *Ttk* knockout in vivo model.**

The carotid arteries of control and *Ttk^ΔSMC^* mice were subjected to sham or wire injury operations. The mice were euthanized, and the carotid arteries were harvested on day 28 post-surgery for subsequent experiments. A) The lysates of carotid arteries were immunoprecipitated with anti-N-cadherin antibodies, and the precipitates were analysed using immunoblotting with anti-p120 and anti-β-catenin antibodies. B) Representative western blotting and quantification of nuclear p120 and β-catenin protein levels in the carotid arteries. Protein levels were normalised to H3 levels (*n* = 6). C) The lysates of carotid arteries were immunoprecipitated with anti-SRF antibodies, and the precipitates were analysed using immunoblotting with anti-MYOCD antibodies. Data are presented as the mean ± SEM; one‐way ANOVA.


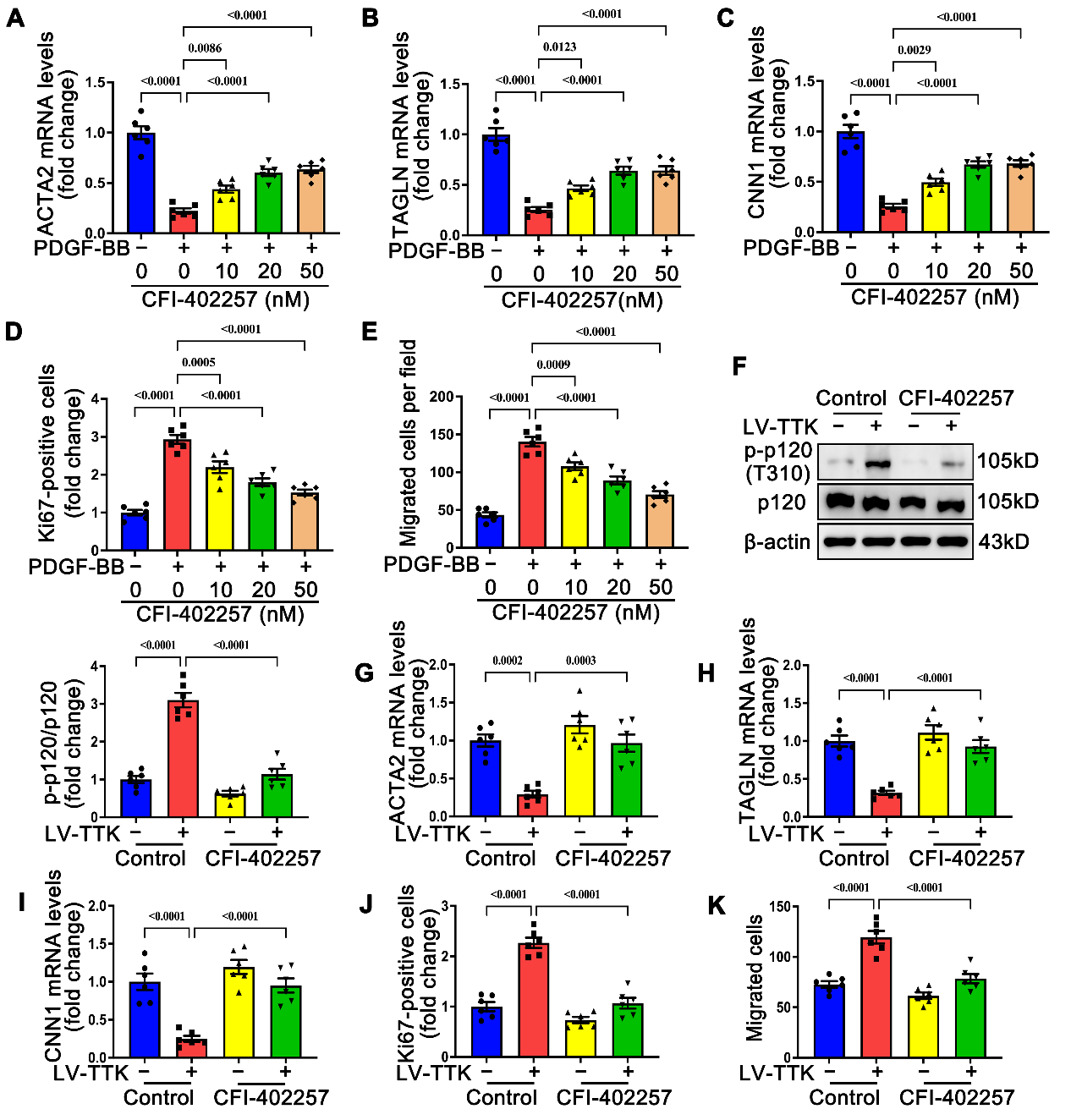


**Figure S11. Effects of TTK inhibitor on the phenotypic switching of VSMCs in vitro.**

A–C) Relative mRNA levels of ACTA2 (A), TAGLN (B), and CNN1 (C) in VSMCs stimulated with different concentrations of CFI-402257 (0, 10, 20, and 50 nM) in the presence or absence of PDGF-BB (20 ng/mL) (*n* = 6). D,E) Quantification analysis of Ki67 immunofluorescence staining D) and transwell assay results E) of VSMCs stimulated with different concentrations of CFI-402257 (0, 10, 20, and 50 nM) in the presence or absence of PDGF-BB (20 ng/mL) (*n* = 6). F) Representative western blotting and quantification of p-p120 (T310) in VSMCs transfected with vector or LV-TTK and treated with or without 50 nM CFI-402257 (*n* = 6). G-I) Relative mRNA levels of ACTA2 (G), TAGLN (H), and CNN1 (I) in VSMCs transfected with vector or LV-TTK and treated with or without 50 nM CFI-402257 (*n* = 6). J,K) Quantification of Ki67 immunofluorescence staining J) and transwell assay results K) of VSMCs transfected with vector or LV-TTK and treated with or without 50 nM CFI-402257 (*n* = 6). Data are presented as the mean ± SEM; one‐way ANOVA.


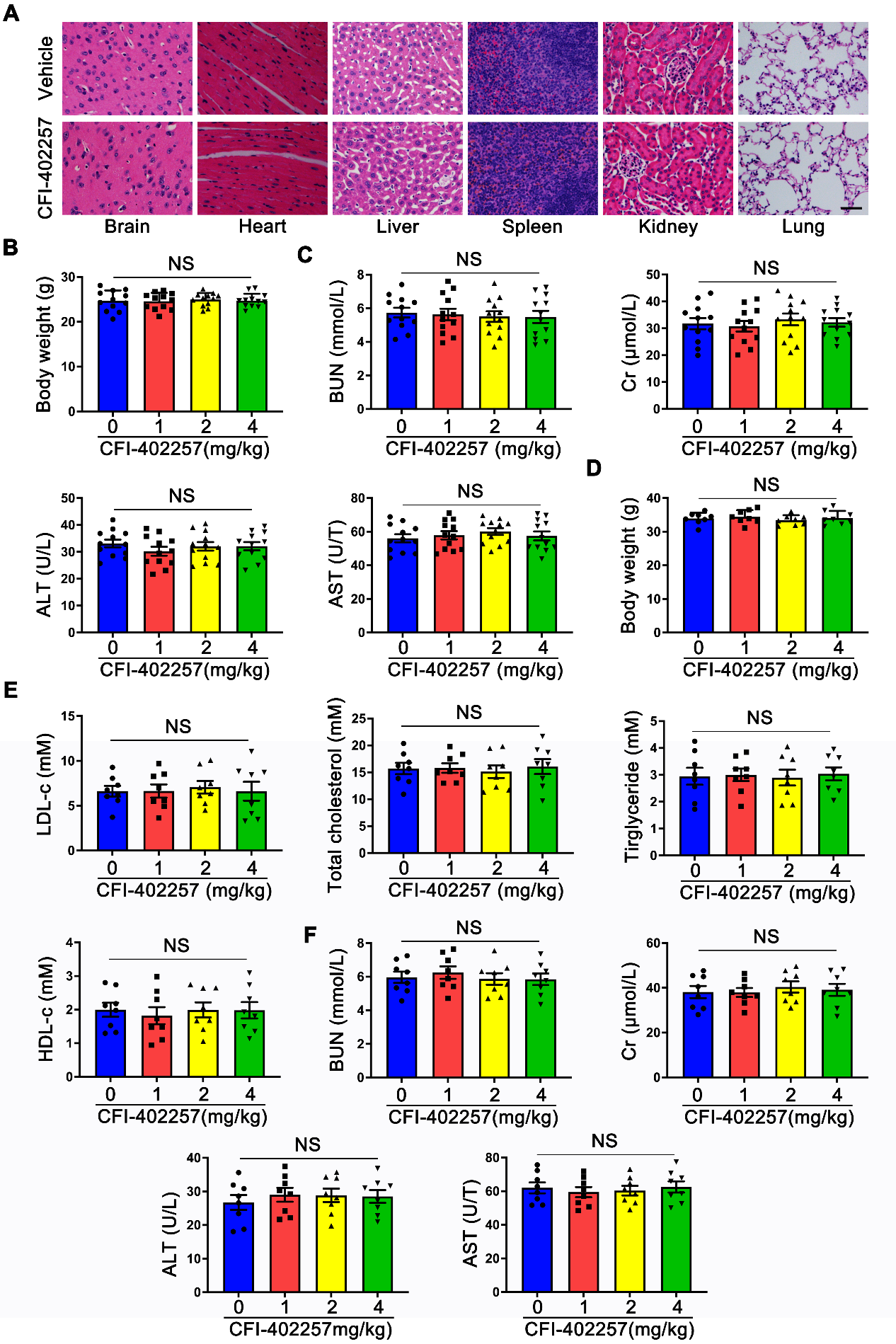


**Figure S12. Administration of TTK inhibitor does not exert systemic toxicity or organ injuries in the mouse models.**

Carotid wire injury mouse model or high-fat diet (HFD)-fed *ApoE^−/−^* mice were administered with CFI-402257 (1, 2, or 4 mg/kg body weight) or vehicle.

A) Representative images of HE staining of the brain, heart, liver, spleen, kidney, and lung tissue sections from C57 mice administered with vehicle or CFI-402257 (4 mg/kg body weight). Scale bar = 50 μm. B) Body weight of the carotid wire injury mouse model treated with CFI-402257 (*n* = 12). C) Blood biochemical parameters, such as blood urea nitrogen (BUN), creatinine (Cr), alanine aminotransferase (ALT), and aspartate aminotransferase (AST) in the carotid wire injury mouse model treated with CFI-402257 (*n* = 12). D–E) Body weight D), serum low-density lipoprotein-cholesterol (LDL-c), total cholesterol, triglyceride, and high-density lipoprotein-cholesterol (HDL-c) levels E) in *ApoE^−/−^* mice treated with CFI-402257 (*n* = 8). F) Blood biochemical parameters, such as BUN, Cr, ALT, and AST in *ApoE^−/−^* mice treated with CFI-402257 (*n* = 8). Data are presented as the mean ± SEM; one‐way ANOVA. NS, non-significant.

**Supplementary tables**

**Table S1. Clinical Characteristics of the patients**

| Characteristic | Normal  (N=14) | Patients  (N=14) |
| --- | --- | --- |
| Age, years | 60±2 | 59±2 |
| Gender (male) | 10 (71.4) | 12 (85.7) |
| Hypertension | 5 (35.7) | 8 (57.1) |
| Diabetes | 3 (21.4) | 3 (21.4) |
| Smoking | 7 (50) | 9 (64.3) |
| TC, mM | 4.46±0.28 | 4.86±0.32 |
| TG, mM | 1.52±0.17 | 1.61±0.22 |
| HDL-c, mM | 1.30±0.10 | 1.42±0.13 |
| LDL-c, mM | 2.59±0.25 | 2.46±0.22 |

Data of age, TC, TG, HDL cholesterol and LDL cholesterol displayed as mean ± SEM (standard error of mean) in each group. Data of gender, hypertension, diabetes and smoking were presented as the occupation % in each group. TC: total cholesterol; TG: triglyceride; HDL-c: high density lipoprotein cholesterol; LDL-c: low density lipoprotein cholesterol.

**Table S2. Primers used for genotyping of murine strains.**

| Primers | Sequence (5’-3’) | Product size |
| --- | --- | --- |
| *ApoE* | GCCTAGCCGAGGGAGAGCCG | WT: 155bp Mutant: 245bp |
|  | TGTGACTTGGGAGCTCTGCAGC |  |
|  | GCCGCCCCGACTGCATCT |  |
| *Ttk flox* | GGAGATATAGTGGAAAGTTACCAAGTG | WT: 290bp Mutant: 391bp |
|  | ATATACTCTACTAGAGCTTTGCTCCCG |  |
| *Myh11-CreER^T2^* | TGACCCCATCTCTTCACTCC | WT: 180bp Mutant: 287bp |
|  | AGTCCCTCACATCCTCAGGTT |  |
| *Myh11^WT^* | CAGCCAACTTTACGCCTAGC |  |
|  | TCTCAAGATGGACCTAATACGG |  |
| *Rosa26^tdTomato^* | CTGTTCCTGTACGGCATGG | WT: 297bp Mutant: 196bp |
|  | GGCATTAAAGCAGCGTATCC |  |
| *Rosa26^WT^* | AAGGGAGCTGCAGTGGAGTA |  |
|  | CCGAAAATCTGTGGGAAGTC |  |

**Table S3. Primers Used for qRT-PCR Analysis.**

| Gene | Forward (5 ́-3 ́) | Reverse (5 ́-3 ́) |
| --- | --- | --- |
| Mouse CEP55 | CCTAGTAGCTCCAAGTCAGACA | ACCTTAGGTGGTCTTTGAGTCTC |
| Mouse TTK | TGCACGCCATAATCAACCCTG | CATGCACTCACTTTTGCAGTTT |
| Mouse NCAPH | TGGCCTCCCCTAACAGGAATA | GCCGAAAGCATTCTTAGTAGTGA |
| Mouse ACTA2 | GTCCCAGACATCAGGGAGTAA | TCGGATACTTCAGCGTCAGGA |
| Mouse TAGLN | CAACAAGGGTCCATCCTACGG | ATCTGGGCGGCCTACATCA |
| Mouse CNN1 | TCTGCACATTTTAACCGAGGTC | GCCAGCTTGTTCTTTACTTCAGC |
| Mouse β-actin | GGCTGTATTCCCCTCCATCG | CCAGTTGGTAACAATGCCATGT |

**Table S4. Sequence of siRNAs.**

| Gene | Sequence |
| --- | --- |
| TTK siRNA-1 | GTCCATCATCAACACCTTA |
| TTK siRNA-2 | GAGCTTTGCTCGAATACAA |
| si-CEP55 | GGTCAAGATATAGCTCTTC |
| si-NCAPH | ACACACAGATTACAGAACA |
| IRF1 siRNA-1 | GGACATTGGGATAGGCATA |
| IRF1 siRNA-2 | GCACCACTGATCTGTATAA |
| E2F4 siRNA-1 | CATCGGTCTGATCGAGAAGAA |
| E2F4 siRNA-2 | CGGTCTGATCGAGAAGAAATC |
| C/EBPβ siRNA-1 | CACCCTGCGGAACTTGTTCAA |
| C/EBPβ siRNA-2 | AGCTGAGCGACGAGTACAAGA |
